# Patchouli alcohol attenuates the cognitive deficits in a transgenic mouse model of Alzheimer’s disease via modulating neuropathology and gut microbiota through suppressing C/EBPβ/AEP pathway

**DOI:** 10.1186/s12974-023-02704-1

**Published:** 2023-01-30

**Authors:** Qing-Qing Xu, Zi-Ren Su, Wen Yang, Mei Zhong, Yan-Fang Xian, Zhi-Xiu Lin

**Affiliations:** 1 School of Chinese Medicine, Faculty of Medicine, The Chinese University of Hong Kong, Shatin, N.T., Hong Kong SAR, People’s Republic of China; 2 Guangdong Provincial Key Laboratory of New Drug Development and Research of Chinese Medicine, Mathematical Engineering Academy of Chinese Medicine, Guangzhou University of Chinese Medicine, Guangzhou, 510006 China; 3 Hong Kong Institute of Integrative Medicine, The Chinese University of Hong Kong, Shatin, N.T., Hong Kong SAR, People’s Republic of China; 4 Li Dak Sum Yip Yio Chin R&D Centre for Chinese Medicine, The Chinese University of Hong Kong, Shatin, N.T., Hong Kong SAR, People’s Republic of China

**Keywords:** Alzheimer’s disease, Patchouli alcohol, TgCRND8, Neuropathology, Gut microbiota, C/EBPβ/AEP pathway

## Abstract

**Background:**

Alzheimer’s disease (AD) is a chronic neurodegenerative disease characterized by progressive cognitive dysfunctions and behavioral impairments. Patchouli alcohol (PA), isolated from *Pogostemonis Herba*, exhibits multiple pharmacological properties, including neuroprotective effects. This study aimed to investigate the therapeutic effects of PA against AD using the TgCRND8 transgenic AD mouse model, and to explore the underlying mechanisms targeting CCAAT/enhancer-binding protein β/asparagine endopeptidase (C/EBPβ/AEP) signaling pathway.

**Methods:**

After genotyping to confirm the transgenicity, drug treatments were administered intragastrically once daily to 3-month-old TgCRND8 mice for 4 consecutive months. Several behavioral tests were applied to assess different aspects of neurological functions. Then the brain and colon tissues were harvested for in-depth mechanistic studies. To further verify whether PA exerts anti-AD effects via modulating C/EBPβ/AEP signaling pathway in TgCRND8 mice, adeno-associated virus (AAV) vectors encoding CEBP/β were bilaterally injected into the hippocampal CA1 region in TgCRND8 mice to overexpress C/EBPβ. Additionally, the fecal microbiota transplantation (FMT) experiment was performed to verify the potential role of gut microbiota on the anti-AD effects of PA.

**Results:**

Our results showed that PA treatment significantly improved activities of daily living (ADL), ameliorated the anxiety-related behavioral deficits and cognitive impairments in TgCRND8 mice. PA modulated the amyloid precursor protein (APP) processing. PA also markedly reduced the levels of beta-amyloid (Aβ) _40_ and Aβ_42_, suppressed Aβ plaque burdens, inhibited tau protein hyperphosphorylation at several sites and relieved neuroinflammation in the brains of TgCRND8 mice. Moreover, PA restored gut dysbiosis and inhibited the activation of the C/EBPβ/AEP signaling pathway in the brain and colon tissues of TgCRND8 mice. Interestingly, PA strikingly alleviated the AD-like pathologies induced by the overexpression of C/EBPβ in TgCRND8 mice. Additionally, the FMT of fecal microbiota from the PA-treated TgCRND8 mice significantly alleviated the cognitive impairments and AD-like pathologies in the germ-free TgCRND8 mice.

**Conclusion:**

All these findings amply demonstrated that PA could ameliorate the cognitive deficits in TgCRND8 mice via suppressing Aβ plaques deposition, hyperphosphorylation of tau protein, neuroinflammation and gut dysbiosis through inhibiting the activation of C/EBPβ/AEP pathway, suggesting that PA is a promising naturally occurring chemical worthy of further development into the pharmaceutical treatment of AD.

## Introduction

Alzheimer’s disease (AD), a chronic neurodegenerative disease characterized by progressive cognitive dysfunctions and behavioral impairments, accounts for approximately 60–70% of all dementia cases [1]. Clinically, AD usually causes memory loss, mood changes, social withdrawal, and declines in thinking and behavioral skills. No cure for AD is available at present, and current therapeutics in the clinic can only provide AD patients with temporary symptomatic relief [2, 3]. Such a dilemma for AD treatment may be partly attributed to the complex pathogenesis of the disease. Despite its multifactorial etiopathogenesis, two neuropathological hallmarks in the AD brain have been well recognized, i.e., neurofibrillary tangles (NFTs) composed mainly of hyperphosphorylated tau protein, and amyloid plaques principally consisting of beta-amyloid (Aβ) peptide [4]. Other pathological features, such as neuroinflammation, gut dysbiosis, oxidative stress, synaptic dysfunctions and neuronal degeneration, are also believed to play important roles in the disease progression [5].

Accumulating evidence suggests that gut dysbiosis is closely associated with AD pathology via the microbiota–gut–brain axis [6]. The microbiota–gut–brain axis is a bidirectional communication system between the central nervous system (CNS) and the gastrointestinal tract via constant communication among neural, immune, endocrine, and metabolic pathways [7]. Previous studies have demonstrated that the composition and diversity of the gut microbiota are altered in AD patients, when compared with the cognitively normal controls [8–10]. In addition, these different gut microbiota taxa are related to cognitive and neuropsychiatric symptoms in AD [9]. Abnormal gut microbiota has been implicated in AD pathogenesis as it is associated with the production of neurotransmitter-like products, the formation of amyloid plaques, and the pro-inflammatory response. Neurotransmitters and neurotoxic substances produced by certain types of microbiotas can enter the brain via the microbiota–gut–brain axis to further exert detrimental effects on cognitive functions [11]. In line with the clinical findings, gut dysbiosis was also observed in the AD animal models. The gut microbiota diversity in the APP/PS1 transgenic AD mouse model decreased with age. The gut microbiota of the APP/PS1 mice was compositionally different from that of wild-type littermates, specifically with decreased *Prevotella* abundance and increased abundances of *Helicobacteraceae* and *Desulfovibrionaceae* at the family level and *Odoribacter* and *Helicobacter* at the genus level in the APP/PS1 mice. The cognitive impairments and the hippocampal amyloid plaque burden were correlated with this particular gut microbiome state [12]. In a separate study, Brandscheid et al*.* [13] found that the relative abundances of *Firmicutes* and *Clostridium leptum* were increased and the amount of *Bacteroidetes* was decreased in the 5 × FAD mice. Interestingly, colonization of germ-free APP/PS1 transgenic mice with microbiota from the wild-type mice ameliorated cerebral amyloid pathology, while colonization of microbiota from conventionally raised APP/PS1 mice accentuated cerebral Aβ burden [14]. On the other hand, cognitive impairments were observed in patients with gastrointestinal (GI) diseases, such as irritable bowel syndrome (IBS) and inflammatory bowel disease (IBD) [15], suggesting that gut microbiota disturbance-mediated GI inflammation promotes the occurrence of cognitive impairments.

Asparagine endopeptidase (AEP, gene name LGMN), known as δ-secretase, plays a critical role in AD pathogenesis. AEP facilitates Aβ production and promotes tau hyperphosphorylation and aggregation by cleaving amyloid precursor protein (APP) and tau. Depleting AEP from Tau P301S or 5 × FAD transgenic mice significantly ameliorates tau pathologies and Aβ deposition, thereby ameliorating synaptic dysfunctions and cognitive deficits in both mouse models [16, 17]. Additionally, as a selective AEP inhibitor, δ-secretase inhibitor 11 was demonstrated to diminish AD pathologies, leading to the improvement of cognitive function in a sporadic AD model [18]. CCAAT/enhancer-binding protein beta (C/EBPβ), an inflammation-regulated transcription factor, modulates the transcription and protein level of AEP in the brain in an age-dependent manner. In addition to regulating the pro-inflammatory process in astrocytes and microglia, C/EPBβ itself increases in response to Aβ [19] and pro-inflammatory stimuli, such as IL-1, IL-6 and TNFα [20, 21], all of which are markedly elevated in the AD patients [22–25]. Overexpression of C/EBPβ increased AEP expression and accelerated the AD pathologies, leading to the aggravation of cognitive deficits in young 3 × Tg mice; and conversely, depletion of C/EBPβ in old 5 × FAD or 3 × Tg mice down-regulated AEP and diminished the pathological features, ultimately ameliorating cognitive dysfunctions in these AD experimental models [26]. Thus, C/EBPβ/AEP signaling pathway is closely associated with AD pathologies.

**Fig. 1:**
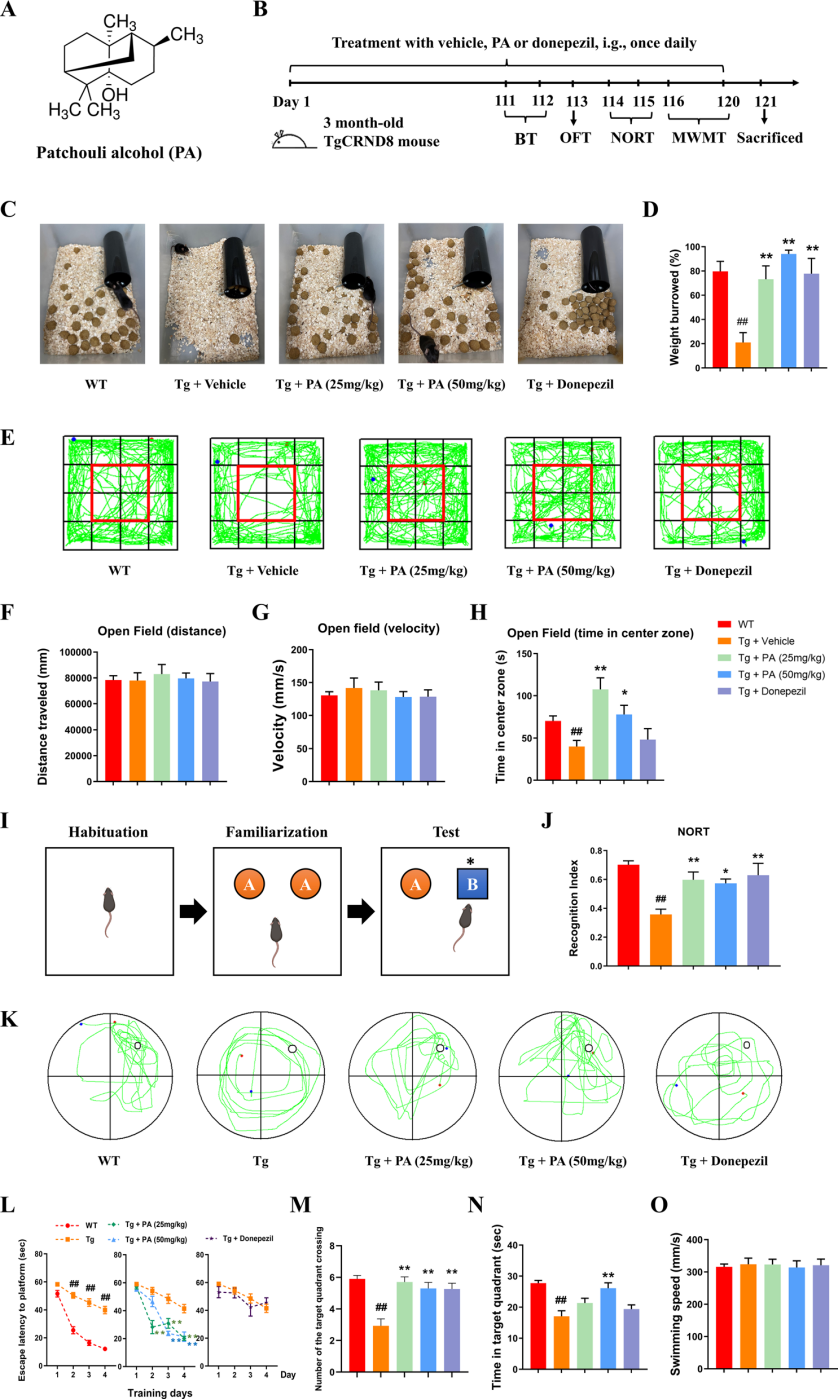
Effects of PA on behavioral impairments and cognitive deficits in TgCRND8 mice. **A** The chemical structure of PA; **B** experimental design and treatment schedule for evaluating the neuroprotective effects of PA on TgCRND8 transgenic mice; **C** representative image of burrowing test overnight; **D** weight burrowed overnight in the burrowing test; **E** representative images of the movement paths in OFT; **F** distance traveled in OFT; **G** movement velocity in OFT; **H** time spent in the center zone in OFT; **I** experimental design of NORT; **J** Recognition Index in NORT; **K** representative images of the swimming paths of mice in the MWMT probe test; **L** escape latency to platform during training days in MWMT; **M** number of the target quadrant crossing in the MWMT probe test; **N** the time spent in the target quadrant in the MWMT probe test; **O** swimming speed in the MWMT probe test. Data were expressed as the mean ± SEM (*n* = 9–10). ^##^*p* < 0.01 compared with the WT group; *p < 0.05 and **p < 0.01 compared with the Tg + vehicle group

Due to the absence of effective treatment, there is an urgent need to discover novel therapeutics for AD patients. Patchouli alcohol (PA, the chemical structure shown in Fig. 1A), one of the main biologically active ingredients isolated from a common Chinese herb *Pogostemonis Herba*, is known to exhibit multiple beneficial pharmacological properties [27, 28], including neuroprotective effects, for various neurological diseases [29]. Our previous studies have demonstrated that PA possesses extensive anti-inflammatory activities [30] and ameliorates cognitive impairments in a well-recognized sporadic AD model induced by streptozotocin [31]. PA has also recently been shown to exert neuroprotective effects against AD in the APP/PS1 mouse model [32]. In addition, accumulating evidence suggests that PA can modulate gut microbiota and exhibit promising protective effects on GI diseases [33]. However, the in vivo efficacy of PA on gut microbiota, tau pathologies and neuroinflammation in familial AD remains unexplored and warrants in-depth investigation.

As an early-onset transgenic AD model, TgCRND8 mice encode a double mutant form of human APP 695 with the Swedish mutation (KM670/671NL) and Indiana mutation (V717F), and overexpress mutant human APP at levels approximately fivefold higher than endogenous murine APP. This model has a rapid onset of cognitive impairments and extracellular thioflavin S-positive Aβ deposits at 3 months of age, with dense-core Aβ plaques and neuritic pathology appearing from 5 months of age [34]. Moreover, tau is hyperphosphorylated and aggregated in the neocortex and hippocampus at 7–12 months of age [35]. Apart from Aβ plaque deposition and abnormal tau processing, microglia activation and robust astrogliosis appear simultaneously with Aβ deposition at 13–14 weeks of age, closely resembling neuroinflammation in the human AD brain [36]. Neuronal Loss, synaptic dysfunctions and abnormal long-term potentiation (LTP) are also observed in TgCRND8 mice [37–39]. Thus, the TgCRND8 transgenic mouse model is an ideal and accurate experimental tool for evaluating the potential efficacy of a therapy for AD, and elucidating its underlying anti-AD molecular mechanisms.

In this study, we aimed to investigate the therapeutic effects of PA on AD using the TgCRND8 transgenic AD mouse model, and to explore the underlying molecular mechanisms targeting C/EBPβ/AEP signaling pathway. We also aimed to unravel the relationship between the modulation of gut microbiota and the anti-AD effects of PA in TgCRND8 transgenic mice.

## Materials and methods

### Chemicals and reagents

PA (purity ≥ 98% by gas chromatography analysis) was isolated and purified in the laboratory of Prof. Ziren SU at Guangzhou University of Chinese Medicine, Guangdong Province, China. Donepezil hydrochloride (Cat. No.: PHR1584-1G), neomycin (Cat. No.: sc-3573A), ampicillin (Cat. No.: sc-202951) and metronidazole (Cat. No.: sc-204805) were purchased from Sigma-Aldrich (St. Louis, USA). All other chemicals and reagents used were of analytical grade.

### Animals

Since gender differences in AD-like pathogenesis were observed in TgCRND8 transgenic mice in our preliminary experiments (data not shown), only age-matched male TgCRND8 (Tg) mice and wild-type (WT) C57BL/6 mice were used in this study. Mice were bred on a hybrid C3H/He-C57BL/6 background [34] in the Run Run Shaw Science Building, The Chinese University of Hong Kong (CUHK). Mice had free access to food and water ad libitum and were housed in a 12:12 h light/dark cycle at 20–24 °C with 40–60% humidity. All animal experiments were approved by the Animal Experimentation Ethics Committee of CUHK (Ref. No. 20/254/NSF). The utmost efforts were made to minimize the suffering and distress of the mice induced by the experimental procedures.

### Polymerase chain reaction (PCR) for genotyping

All mice were genotyped for the expression of APP transgene as previously described [40, 41] when they reached one month of age. In brief, DNA was extracted from the ear of a mouse. The APP transgene was identified by a PCR reaction using the APP gene-specific primers. Subsequently, a visible amount of the ear tissue was incubated in 200 µL non-SDS tissue digesting buffer (500 mM KCl, 100 mM Tris–HCl, 0.1 mg/mL gelatin, 0.45% NP-40 and 0.45% Tween 20, pH = 8.3) containing 5 µL proteinase K (Cat. V900887, Sigma) at 55 °C overnight. The samples were then heated at 98 °C for 10 min to inactivate proteinase K. After centrifugation at 13,000 rpm for 1 min, the supernatants were collected for PCR reaction. PCR was performed on a Light Cycler thermal cycler system (Bio-Rad, United States) using TaKaRa Taq™ (Cat. No.: R001A, TAKARA) and gene-specific primers. The APP gene-specific primers used were as follows: forward, 5′-TGTCCAAGATGCAGCAGAACGGCTAC-3′ and revised, 5′-GGCCGCGGAGAAATGAAGAAACGCCA-3′. The reactions were run at 95 °C for 5 min, followed by 45 cycles at 95 °C for 30 s, 54 °C for 40 s, 72 °C for 80 s and 72 °C for 10 min. Thereafter, the PCR products were separated on 1% agarose gel together with loading buffer (Cat. GR0206-5, Shanghai Generay Biotech Co, Ltd), and then visualized under UV light (365 wavelength) for 1 min. The mice with APP transgene expression were identified as transgenic mice.

### Animal grouping and drug administration

Experiment 1: To investigate the effects of PA on the TgCRND8 mice, 3-month-old male WT and Tg mice were assigned into five groups (*n* = 10): WT group, Tg + vehicle group, Tg + PA (25 and 50 mg/kg) groups and Tg + donepezil (5 mg/kg) group. Donepezil was used as the positive control as previously described [40, 41]. PA was dissolved in 1% Tween-80, and donepezil was dissolved in distilled water. PA or donepezil was administered intragastrically once daily to mice for 4 consecutive months, while the WT group and the Tg + vehicle group received the same volume of vehicle (1% Tween-80) for the same duration. At the end of drug treatment, behavioral tests, including the burrowing test (BT), the open field test (OFT), the novel object recognition test (NORT) and the Morris water maze test (MWMT), were conducted to assess the neurological functions in mice. After behavioral tests, the mice were killed under deep anesthesia, and the brain and colon tissues were collected for mechanistic evaluations. The experimental schedule for Experimental 1 is shown in Fig. 1B.

Experiment 2: To further identify the potential mechanisms underlying the effects of PA on the TgCRND8 mice, 3-month-old male WT and Tg mice were assigned into five groups (*n* = 8): WT + Overexpression (OE)-control group, Tg + OE-control group, Tg + OE-CEBP/β group and Tg + OE-CEBP/β + PA group. Drug treatment was initiated one day after the stereotaxic injection and lasted till the killing of the mice. PA at a dose of 50 mg/kg was administered intragastrically once daily to mice for 4 consecutive months, while the same volume of vehicle (1% Tween-80) was given to the mice in the WT + OE-control group, the Tg + OE-control group and the Tg + OE-CEBP/β group for the same duration. At the end of drug treatment, behavioral tests, including the OFT, the NORT and the MWMT, were conducted to assess the neurological functions of the mice. The experimental schedule for Experimental 2 is shown in Fig. 6B.

Experiment 3: To further examine the involvement of gut microbiota in the anti-AD effects of PA, fecal microbial transplantation (FMT) treatment was conducted. Firstly, 3-month-old male WT mice (*n* = 8) and Tg mice (*n* = 32) received antibiotic treatment to establish a pseudo-germ-free mouse model. Mice were administered intragastrically once daily (0.2 mL/mouse) with  an antibiotic cocktail (ABX) containing 1.25 g/L neomycin, 2.5 g/L ampicillin, and 2.5 g/L metronidazole for 5 consecutive days to remove indigenous gut microbes. After the antibiotic treatment, mice were randomly assigned into five groups (*n* = 8): WT group, Tg + vehicle group, Tg + PA-FMT group, Tg + WT-FMT group and Tg + Tg-FMT group. Mice received vehicle (PBS) or the settled suspension of feces (50 mg/mL in PBS) from 7-month-old mouse donors (obtained from Experiment 1, including 7-month-old WT mice, Tg mice and Tg mice with 50 mg/kg PA treatment) by oral gavage once daily (0.2 mL/mouse) for 3 consecutive months. After conducting behavioral tests including OFT, NORT and MWMT, the mice were killed under anesthesia. The experimental design and schedule are shown in Fig. 7A, B.

### Stereotaxic injection in the mouse hippocampus

Adeno-associated virus (AAV) vectors encoding CEBP/β were used to obtain long-term expression of CEBP/β in the mouse brain. AAV2/9-CMV-CEBP/β-3 × flag-ZsGreen (titer: 1.2 × 10^12^ vector genome (vg)/mL) and AAV2/9-ZsGreen (titer: 1.3 × 10^12^ vg/mL, used as control) were generated by Hanbio Biotechnology Co., Ltd (Shanghai, China) and stored at − 80 °C until use. AAV vectors were bilaterally injected into the hippocampal CA1 region. Briefly, after being anaesthetized by intraperitoneal injection of ketamine (100 mg/kg) and xylazine (10 mg/kg), 3-month-old mice were fixed in a prone position on the stereotaxic apparatus (Stoelting, Wood Dale, IL, USA). After a sagittal incision in the midline of the scalp, small burr holes (1 mm in diameter) were drilled into the skull. AAV vectors were bilaterally injected into the hippocampal CA1 region (in a volume of 1 μL/ventricle) at a rate of 0.2 μL/min. The following stereotaxic coordinates were applied for hippocampal injection according to the brain atlas of Paxinos and Franklin [42]: 2.0 mm posterior to bregma, ± 1.2 mm lateral to sagittal suture and 2.0 mm ventral from the surface of the skull. After recovery from anesthesia, mice were returned to cages and allowed free access to food and water.

### Behavior tests

#### Burrowing test

The burrowing test was used to evaluate activities of daily living (ADL) in mice as previously described [43] with minor modifications. Briefly, a practice run for habituation was conducted on the first day and the burrowing test was performed on the second day. For both the practice run and the test, mice were individually housed in the home cage with a black plastic tube (200 mm long, 75 mm in diameter) overnight. The tube was filled with 200 g of food pellets, and the open end of the tube was raised 30 mm to minimize the risk of the mouse displacing the contents by non-burrowing movements. The tube containing 200 g of food pellets was put into the cage three hours before the dark cycle (at 18:00). The remaining food pellets in the tube were weighed and recorded after 2 h (at 20:00) and overnight. The percentage of burrowed food pellets on the second day was exhibited in the results.

#### Open field test (OFT)

The OFT was conducted to assess the locomotor activity and the anxiety level of the mice, as described in our previous study [44]. The mice were placed in the center of an open field (40 cm × 40 cm × 40 cm) with dim light, and allowed to explore freely in the open field for 10 min. A video tracking system (SuperMaze V2.0, China) was applied to record the movement routes, the velocity, and the time exploring in the center zone of each animal. 70% ethanol was used to thoroughly clean the apparatus before use and before subsequent tests to avoid the scent clues left by the previous subject mouse.

#### Novel object recognition test (NORT)

The NORT was conducted to assess the recognition memory of the mice as described in our previous study [44]. The NORT was performed in an open-field arena (40 cm × 40 cm × 40 cm, the same apparatus of OFT) for three consecutive days. On day 1, the mice were allowed to explore freely in the arena (no objects, data used for open field analyses) for 10 min for habituation. On day 2, the mice were placed in the arena with two identical objects (2.5 cm in diameter, 8 cm in height, yellow glass cylinder, defined as object A) and allowed to explore for 10 min. On day 3, one of the object A was replaced with a new color and shape object (2.5 × 3 × 7.8 cm, a cell culture flask filled with crystal violet solution, defined as object B), and the mice were placed in the arena for free exploration for 10 min. 70% Ethanol was used to clean the objects and the apparatus prior to use and before subsequent tests to minimize olfactory clues left by the previous subject mouse. A video tracking system (SuperMaze V2.0 software, China) was applied to record the time of each mouse spent exploring the objects. The exploratory behavior of the animal was defined as directing the nose toward the object at a distance less than or equal to 2 cm. The recognition index was calculated by the equation: [(Time exploring in the novel object)/(Time exploring in the novel object + Time exploring in the familiar object)].

#### Morris water maze test (MWMT)

The MWMT was performed as described in our previous studies [40, 41, 45] to assess the spatial learning and memory of the mice. Briefly, a circular pool (150 cm in diameter) with water at 24 °C was applied as the experimental apparatus. The pool was conceptually and equally divided into four quadrants by placing four different shape labels on the pool. A circular platform with a diameter of 6 cm and a height of 26 cm was submerged 1 cm below the water surface at the midpoint of one quadrant. Mice were trained to find the hidden platform for four consecutive days with three trials per day. Each mouse was gently placed in the water from different release positions and provided a maximum of 60 s to climb onto the hidden platform in each trial. The time to find the hidden platform (escape latency) was recorded in each trial. Mice were manually guided to the submerged platform and allowed to stay for 30 s when they failed to find the platform within 60 s, and the escape latency was recorded as 60 s. On the fifth day, the probe test was performed after removing the hidden platform. The experimental animals were allowed to swim freely for 60 s in each trial. A video tracking system SuperMaze V2.0 (Xinruan, China) was used to record the swimming routines, the numbers of the target quadrant crossing, the time spent in the target quadrant, and the swimming speed.

### Preparation of the brain tissues

Mice under deep anesthesia were killed 24 h after the behavior tests. Five or six mice in each group (six mice in Experiment 1 and five in Experiment 2 and 3) were transcardially perfused with pre-chilled normal saline until the fluid coming out from the right atrium was clear. The hippocampal and cortex tissues were then isolated from the brains on ice and stored at – 80 °C until use. These samples were used for western blot analysis and the enzyme-linked immunosorbent assay (ELISA) kit assay.

For immunofluorescence analysis, the other portion of mice under deep anesthesia were perfused transcardially with pre-chilled saline (0.9%) followed by 4% paraformaldehyde (PFA) [40, 41, 44]. The brains were then removed and kept in 4% PFA overnight at 4 °C. After gradient elution with sucrose, coronal brain sections were sectioned made at 8 µm thick using a cryostat (Leica CM1850, Leica Microsystems GmbH, Wetzlar, Germany). The brain sections were stored at – 80 °C until use.

### Measurement of the levels of Aβ_40_ and Aβ_42_

The levels of Aβ_40_ and Aβ_42_ in the hippocampal tissues were assessed using commercially available ELISA kits (Cat. No. KMB3481 and KMB3441, respectively, Invitrogen, USA) according to the manufacturer’s instructions. In brief, the hippocampal tissues were homogenized in 8× the brain mass of ice-cold lysis buffer (5 M guanidine-HCl/50 mM Tris, pH 8.0) containing a protease inhibitor cocktail (Sigma-Aldrich, USA). The homogenates were mixed with an orbital shaker at room temperature for 4 h and then centrifugated at 16,000×*g* at 4 °C for 20 min. The supernatants were collected and diluted with standard diluent buffer to an appropriate concentration. The diluted samples were added into the wells that precoated with mAb to NH2 terminus of Aβ and then incubated at room temperature for 2 h to bind the antigen. After aspirating the solution thoroughly and washing wells with 1× wash buffer between each step, the mouse Aβ detection antibody solution, the anti-rabbit IgG HRP solution and the stabilized chromogen solution were sequentially added and incubated at room temperature for 1 h, 30 min and 30 min, respectively. The reaction was terminated by adding the stop solution. The absorbance was read at 450 nm within 5 min using a microplate reader (BMG Labtech, Offenburg, Germany). Meanwhile, the protein concentration in the samples was determined using a BCA protein assay kit (Invitrogen, USA). The levels of Aβ_40_ and Aβ_42_ were calculated using the standard curves and expressed as pg/mg protein.

### Measurement of cytokines

The cortex and colon tissues of mice were homogenized in the pre-chilled lysis buffer (Abcam, UK) to prepare 10% (w/v) homogenates. After centrifugation at 10,000×*g* for 15 min at 4 °C, the supernatants were collected and the protein concentration in the supernatants were determined using the BCA assay method. The levels of interleukin-1β (IL-1β), IL-4, IL-6, interferon gamma (IFN-γ) and tumor necrosis factor alpha (TNF-α) were assessed using commercial ELISA kits (IL-1β, IL-4, IL-6 and IFN-γ from Abcam; TNF-α from RayBiotech) according to the manufacturer’s instructions. The levels of IL-1β, IL-4, IL-6, IFN-γ and TNF-α were calculated using the standard curves and expressed as pg/mg protein.

### Western blot analysis

Western blot analysis was performed as previously described [45]. Briefly, the hippocampus and colon tissues were homogenized in the pre-chilled RIPA lysis buffer (Cat. 89901, Thermo Scientific, USA) with 1× protease/phosphatase inhibitor cocktail (Cat. 78442, Thermo Scientific, USA) to prepare 10% (w/v) homogenates. After centrifugation at 13,000 rpm at 4 °C for 20 min, the supernatants were collected. The protein concentrations were determined using the BCA assay method. Equal amounts (30 μg) of denatured protein samples were separated on SDS–polyacrylamide gels, and subsequently transferred to polyvinyl difluoride (PVDF) membranes. After being blocked with 5% non-fat milk for 2 h, the membranes were incubated overnight at 4 °C with primary antibodies, including ADAM-10 (1:500, sc-48400, Santa Cruz), BACE-1 (1:5000, ab108394, Abcam), p-APP (Thr688) (1:1000, 6986S, Cell Signaling Technology), anterior pharynx-defective-1 (APH-1) (1:1000, PRS4001, Sigma), CTFs (1:1000, A8717, Sigma), insulin degrading enzyme (IDE) (1:500, sc-393887, Santa Cruz), Tau (Tau 5) (1:500, sc-58860 Santa Cruz), p-Tau (Thr181) (1:1000, 12885S, Cell Signaling Technology), p-Tau (Thr205) (1:1000, ab254410, Abcam), p-Tau (Ser396) (1:10,000, ab109390, Abcam), p-Tau (Ser404) (1:1000, ab92676, Abcam), BDNF (1:1000, 47808S, Cell Signaling Technology), C/EBPβ (1:500, sc-7962, Santa Cruz), AEP (1:500, sc-133234, Santa Cruz), Tau (N368) (1:1000, ABN1703, Sigma), β-actin (1:1000, sc-69879, Santa Cruz) and GAPDH (1:5000, ab181602, Abcam). After overnight incubation, the membranes were incubated with anti-rabbit or anti-mouse IgG secondary antibody (1:3000, Cell Signaling Technology) at room temperature for 2 h. The protein bands were visualized using ECL reagent (Cat. 34580, Thermo Scientific). Image J software (NIH, Bethesda, MD, USA) was used for the densitometric analysis.

### Immunofluorescence staining

Immunofluorescence staining was performed as previously described [40, 41] with minor modifications. Briefly, after being air-dried at room temperature for 30 min, the brain sections were permeabilized for 20 min using 0.5% Triton X-100, and subsequently blocked in 10% normal donkey serum (Abcam, UK) for 1 h. Then the brain sections were incubated with primary antibodies overnight at 4 °C, including β-amyloid (1:400, Cat. A5213, Sigma), Iba-1 (1:400, Cat. 019-19741, Wako) and GFAP (1:400, Cat. HPA056030, Sigma). On the following day, after being washed with 1× phosphate buffered saline with Tween^®^ 20 (PBST) for three times, the brain sections were incubated with goat anti-mouse IgG H&L (Alexa Fluor^®^ 546) secondary antibody (1:500, A11003, Invitrogen) and donkey anti-rabbit IgG H&L (Alexa Fluor^®^ 488) secondary antibody (1:500, ab150105, Abcam) at 37 °C for 1 h in the dark. Nuclei were counterstained using 4,6-diamidino-2-phenylindole (DAPI) (Cat. S36973, Invitrogen). Images were obtained using a fluorescent microscope (Zeiss, Gottingen, Germany) and analyzed using Image J software.

### Analysis of gut microbiota diversity and composition by 16S rRNA gene sequencing

#### Sample collection and DNA extraction

Fresh fecal samples were collected from 7-month-old mice in Experiment 1 (including mice in the WT group, the Tg + vehicle group, the Tg + PA (50 mg/kg) group and the Tg + donepezil group) and stored at − 80 °C until use. The genomic DNA from fecal samples was extracted using Omega Mag-Bind Soil DNA Kit (M5635-02, Omega, USA) according to the manufacturer’s protocols. DNA concentration was measured by NanoDrop™ 2000 Spectrophotometers (Thermo Fisher, USA), and then further diluted to 1 ng/μL for sequencing using sterile water.

#### Library construction and sequencing

Hypervariant region V3–V4 of the bacterial 16S rRNA gene was amplified with the forward primer 341F (5′-ACTCCTACGGGAGGCAGCA-3′) and reverse primer 806R (5′-GGACTACHVGGGTWTCTAAT-3′) by PCR reaction. PCR reaction system was 30 μL containing 0.2 μM of forward and reverse primers, 10 ng template DNA and 15 μL of Phusion^®^ High-Fidelity PCR Master Mix (New England Biolabs, USA). The PCR reaction consisted of initial denaturation at 98 °C for 1 min, followed by 30 cycles of denaturation at 98 °C for 10 s, annealing at 50 °C for 30 s and elongation at 72 °C for 60 s, with a final 5 min at 72 °C. PCR products were mixed with 1× loading buffer (Containing SYB green) and the same volume of the mixtures was monitored on 2% agarose gels. Samples with the bright main strip between 400 and 450 bp were chosen for further experiments. Then the amplicons were purified with AxyPrepDNA Gel Extraction Kit (AXYGEN) following the manufacturer’s protocol. Sequencing libraries were generated using NEB Next^®^Ultra™DNA Library Prep Kit for Illumina (NEB, USA) according to  the manufacturer’s recommendations at Shanghai Applied Protein Technology Co., Ltd (Shanghai, China). The quality of sequencing library was assessed on the Qubit@ 2.0 Fluorometer (Thermo Scientific, USA) and Agilent Bioanalyzer 2100 system (Agilent Technologies, USA).

#### Data analysis

The sequence analysis was performed by the UPARSE software package using the UPARSE-OTU and UPARSE-OTUref algorithms. The taxonomy of each sequence was analyzed by QIIME software and α-diversity indexes were compared using rarefied data. The principal coordinate analysis (PCoA) plot was implemented by R programming language.

### Statistical analysis

All data were presented as means ± standard error of the mean (mean ± SEM). Group differences in the escape latency in the MWMT training task were analyzed using two-way ANOVA followed by post hoc Bonferroni’s multiple comparison test with repeated measures, with the factors being treatment and training day. The other differences between multiple groups were analyzed using one-way ANOVA followed by Turkey multiple comparison test and differences between two groups were analyzed using *t*-test. A *p* < 0.05 was considered to be statistically significant.

## Results

### Effects of PA on behavioral impairments and cognitive deficits in TgCRND8 mice

#### Effects of PA on ADL in TgCRND8 mice

Burrowing test was applied to determine the ADL skills in TgCRND8 mice. When compared with the WT group, the Tg + vehicle group showed a significant deficit in spontaneous burrowing behavior (*p* < 0.01). The mice in all of the treatment groups, including the Tg + PA (25 mg/kg) group, the Tg + PA (50 mg/kg) group and the Tg + donepezil group, burrowed markedly more weight of food pellets (*p* < 0.01 for all), as compared with the Tg control mice (Fig. 1C, D).

#### Effects of PA on the locomotor activity and the anxiety-related behavior in TgCRND8 mice

As shown in Fig. 1F, G, open field performance showed no significant differences in distance traveled and movement velocity among all groups, indicating that PA did not cause motor coordination deficit. In the OFT, animals with elevated anxiety levels spent less time exploring the center of the chamber. When compared with the WT group, there was a significant reduction in the time exploring the center zone of the chamber (*p* < 0.01) in the Tg + vehicle group (Fig. 1H). The Tg mice treated with PA (25 mg/kg and 50 mg/kg) spent significantly more time exploring the center zone than the mice in the Tg control group (*p* < 0.01 and *p* < 0.05, respectively), indicating that PA could alleviate the anxiety-related behavior deficits in TgCRND8 mice.

#### Effects of PA on the recognition memory in TgCRND8 mice

The NORT was used to assess recognition memory in mice. This test is based on the spontaneous tendency of mice to spend more time exploring a novel object than a familiar one. As shown in Fig. 1I, J, the Tg + vehicle group had a markedly lower recognition index than the WT group (*p* < 0.01), suggesting that Tg mice had an impaired ability to discriminate between familiar and novel objects as compared with the WT mice. The administration of PA (25 mg/kg and 50 mg/kg) effectively increased the recognition index in the Tg mice (*p* < 0.01 and *p* < 0.05, respectively), when compared with the Tg control group (Fig. 1J). In addition, the Tg + donepezil group also had a higher recognition index than the Tg + vehicle group (*p* < 0.01). All these experimental findings demonstrated that PA could improve the recognition memory impairments in TgCRND8 mice.

#### Effects of PA on cognitive and learning functions in TgCRND8 mice

MWMT was used to assess spatial learning and memory in TgCRND8 mice. As shown in Fig. 1L, during the training days, the mice in the Tg + vehicle group spent significantly longer time to find the hidden platform than the WT group (from day 2 to day 4, *p* < 0.01 for 3 days). As compared with the Tg control group, the mice in both the Tg + PA (25 mg/kg) and the Tg + PA (50 mg/kg) groups had a shorter escape latency finding the hidden platform during the training days (PA at the dose of 25 mg/kg: *p* < 0.01 for day 2 to day 4; PA at the dose of 50 mg/kg: *p* < 0.01 for day 3 and day 4). In the probe trial, when compared with the WT group, there were conspicuous reductions in the number of the target quadrant crossing and the time spent in the target quadrant in the Tg + vehicle group (*p* < 0.01 for both) (Fig. 1M, N), demonstrating that Tg mice had cognitive and memory impairments. The administration of all treatments, including PA (25 mg/kg and 50 mg/kg) and donepezil, effectively increased the number of the target quadrant crossing in the Tg mice (*p* < 0.01 for all), when compared with the Tg control group (Fig. 1M). The Tg mice with PA treatment at high dose (50 mg/kg) spent significantly more time in the target quadrant than the mice in the Tg control group (*p* < 0.01) (Fig. 1N). In addition, the administration of PA at low dose (25 mg/kg) and donepezil increased the time spent in the target quadrant in Tg mice but differences were not statistically significant (*p* > 0.05), as compared with Tg control group. There were no statistical differences in the swimming speed among all groups (Fig. 1O). These experimental results indicated that PA could ameliorate learning and memory impairments in TgCRND8 mice.

### Effects of PA on the hyperphosphorylation of tau protein in TgCRND8 mice

**Fig. 2:**
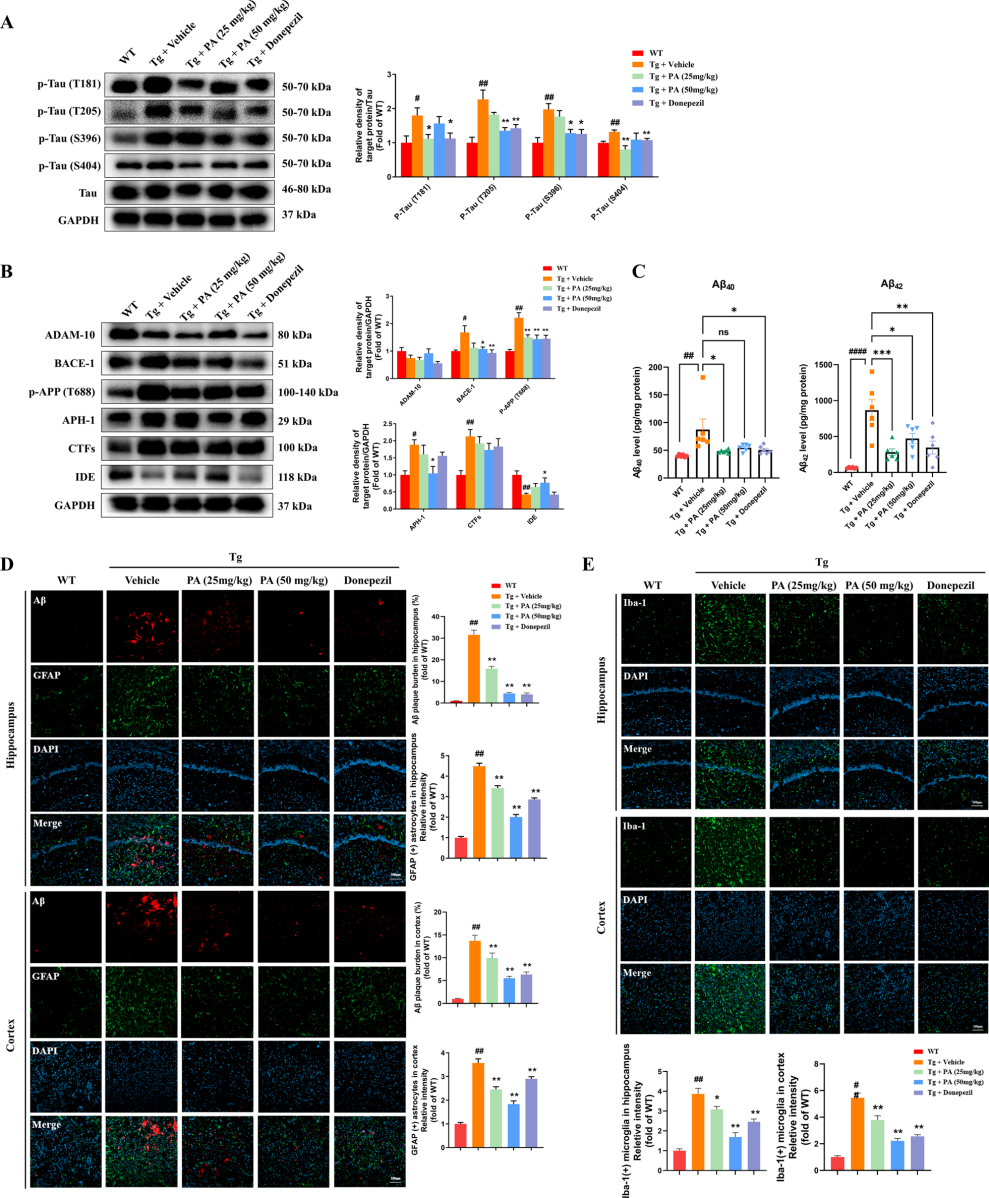
Effects of PA on the hyperphosphorylation of tau protein, Aβ pathology, GFAP-positive astrocytes and Iba-1-positive microglia in TgCRND8 mice. **A** Effects of PA on tau hyperphosphorylation at sites of T181, T205, S396 and S404 in TgCRND8 mice (*n* = 5–6); **B** effects of PA on the APP processing and APP phosphorylation in TgCRND8 mice. Representative western blotting images and quantitative analysis of the protein expressions of ADAM-10, BACE-1, p-APP (Thr 688), APH-1, CTFs and IDE (*n* = 5–6); **C** the levels of Aβ_40_ and Aβ_42_ (*n* = 6); **D** immunofluorescence staining of Aβ and GFAP in the hippocampus and cortex (*n* = 3); **E** immunofluorescence staining of Iba-1 in the hippocampus and cortex (*n* = 3). Data were expressed as the mean ± SEM (*n* = 3–6). ^#^*p* < 0.05, ^##^*p* < 0.01 and ^####^*p* < 0.0001 compared with the WT group; *p < 0.05, **p < 0.01 and ***p < 0.001 compared with the Tg + vehicle group

The potential effects of PA treatment on the phosphorylation of tau protein, the pathological hallmark of AD, were then evaluated. As shown in Fig. 2A, when compared with the WT group, the protein expressions of p-Tau (T181) (*p* < 0.05), p-Tau (T205) (*p* < 0.01), p-Tau (S396) (*p* < 0.01) and p-Tau (S404) (*p* < 0.01) were significantly elevated in the Tg + vehicle group. PA treatment at the lower dose (25 mg/kg) could markedly inhibit the hyperphosphorylation of tau protein at the sites of T181 (*p* < 0.05) and S404 (*p* < 0.01), as compared with the Tg control group, while PA treatment at the higher dose (50 mg/kg) effectively decreased the protein expressions of p-Tau (T205) (*p* < 0.01) and p-Tau (S396) (*p* < 0.05). Moreover, the Tg + donepezil group had lower protein expressions of p-Tau (T181) (*p* < 0.05), p-Tau (T205) (*p* < 0.01), p-Tau (S396) (*p* < 0.05) and p-Tau (S404) (*p* < 0.01) than that of the Tg + vehicle group. These results amply demonstrated that PA could inhibit the hyperphosphorylation of tau protein in TgCRND8 mice.

### Effects of PA on Aβ pathology in TgCRND8 mice

As the formation of amyloid plaques is another pathological hallmark in the AD brain, the effects of PA on Aβ pathology were then detected in TgCRND8 mice. Firstly, to assess the effects of PA on APP processing, elimination and phosphorylation in TgCRND8 mice, the protein expressions of APP α-secretase enzyme ADAM-10, β-secretase enzyme BACE-1, γ-secretase enzyme APH-1, APP C-terminal fragments (CTFs), the major Aβ-degrading enzyme IDE and the phosphorylated APP at the site of Thr688 were measured. As shown in Fig. 2B, when compared with the WT group, the protein expressions of BACE-1 (*p* < 0.05), p-APP (T688) (*p* < 0.01), APH-1 (*p* < 0.05) and CTFs (*p* < 0.01) was significantly accentuated in the Tg + vehicle group, while the protein level of IDE was markedly attenuated (*p* < 0.01). PA treatment at the dose of 50 mg/kg effectively reduced the protein expressions of BACE-1 (*p* < 0.05), APH-1 (*p* < 0.05) and p-APP (T688) (*p* < 0.01) and increased the protein level of IDE (*p* < 0.05), as compared with the Tg control group. Both the PA (25 mg/kg) and donepezil treatment markedly inhibited the hyperphosphorylation of APP at the site of Thr688 (*p* < 0.01 for both), when compared with the Tg control group. The Tg + donepezil group also significantly lowered the expression of BACE-1, when compared with the Tg + vehicle group (*p* < 0.01). There were no significant differences among all groups in the expression of ADAM-10.

Then the effects of PA on the levels of Aβ_40_ and Aβ_42_, the two major Aβ peptides preceding Aβ plaque deposition, were measured. As shown in Fig. 2C, the levels of Aβ_40_ and Aβ_42_ in the Tg + vehicle group were significantly increased (*p* < 0.01 and *p* < 0.0001, respectively), as compared with the WT group. PA treatment at lower dose (25 mg/kg) could markedly inhibit the elevated levels of Aβ_40_ (*p* < 0.05) and Aβ_42_ (*p* < 0.001), when compared with the Tg control group, while PA treatment at higher dose (50 mg/kg) markedly reduced the level of Aβ_42_ (*p* < 0.05). Additionally, the Tg + PA (50 mg/kg) group lowered Aβ_40_ level than the Tg + vehicle group, although not statistically significant. The administration of donepezil significantly decreased the levels of Aβ_40_ (*p* < 0.05) and Aβ_42_ (*p* < 0.01), as compared with the Tg control group. All these findings indicated that PA could reduce the elevated levels of Aβ_40_ and Aβ_42_ in TgCRND8 mice.

The immunofluorescence staining results showed that when compared with the WT group, Aβ plaque burdens were significantly increased in both the hippocampus (*p* < 0.01) and the cortex (*p* < 0.01) in the Tg control group. All of the treatments, including PA (25 and 50 mg/kg) and donepezil, markedly reduced the elevated Aβ plaque burdens in both the hippocampus (*p* < 0.01 for all) and the cortex (*p* < 0.01 for all), as compared with the Tg + vehicle group (Fig. 2D).

### Effects of PA on GFAP-positive astrocytes and Iba-1-positive microglia in the hippocampus and cortex of TgCRND8 mice

The immunofluorescence staining results showed that the Tg control group had a significantly higher density of GFAP-positive astrocytes in both the hippocampus (*p* < 0.01) and the cortex (*p* < 0.01) than the WT group, and the increased density was effectively attenuated upon PA treatment (25 and 50 mg/kg) in both the hippocampus (*p* < 0.01 for both) and the cortex (*p* < 0.01 for both). Donepezil treatment also significantly decreased the density of GFAP-positive astrocytes in both of the hippocampus (*p* < 0.01) and the cortex (*p* < 0.01), as compared with the Tg control group (Fig. 2D).

Similarly, a significant increase of Iba-1-positive microglia density was observed in both the hippocampus (*p* < 0.01) and the cortex (*p* < 0.01) in the Tg + vehicle group, as compared with the WT group. Both of the Tg + PA (25 mg/kg) group and the Tg + PA (50 mg/kg) group had marked lower density of Iba-1-positive microglia than that of the Tg control group in both the hippocampus (*p* < 0.05 and *p* < 0.01, respectively) and the cortex (*p* < 0.01 for both). Donepezil treatment also markedly reduced the microglia density in the hippocampus (*p* < 0.01) and cortex (*p* < 0.01) of the mice, when compared with the Tg control group (Fig. 2E).

### Effects of PA on pro-inflammatory cytokines, anti-inflammatory cytokines and BDNF expression in the brains of TgCRND8 mice

**Fig. 3:**
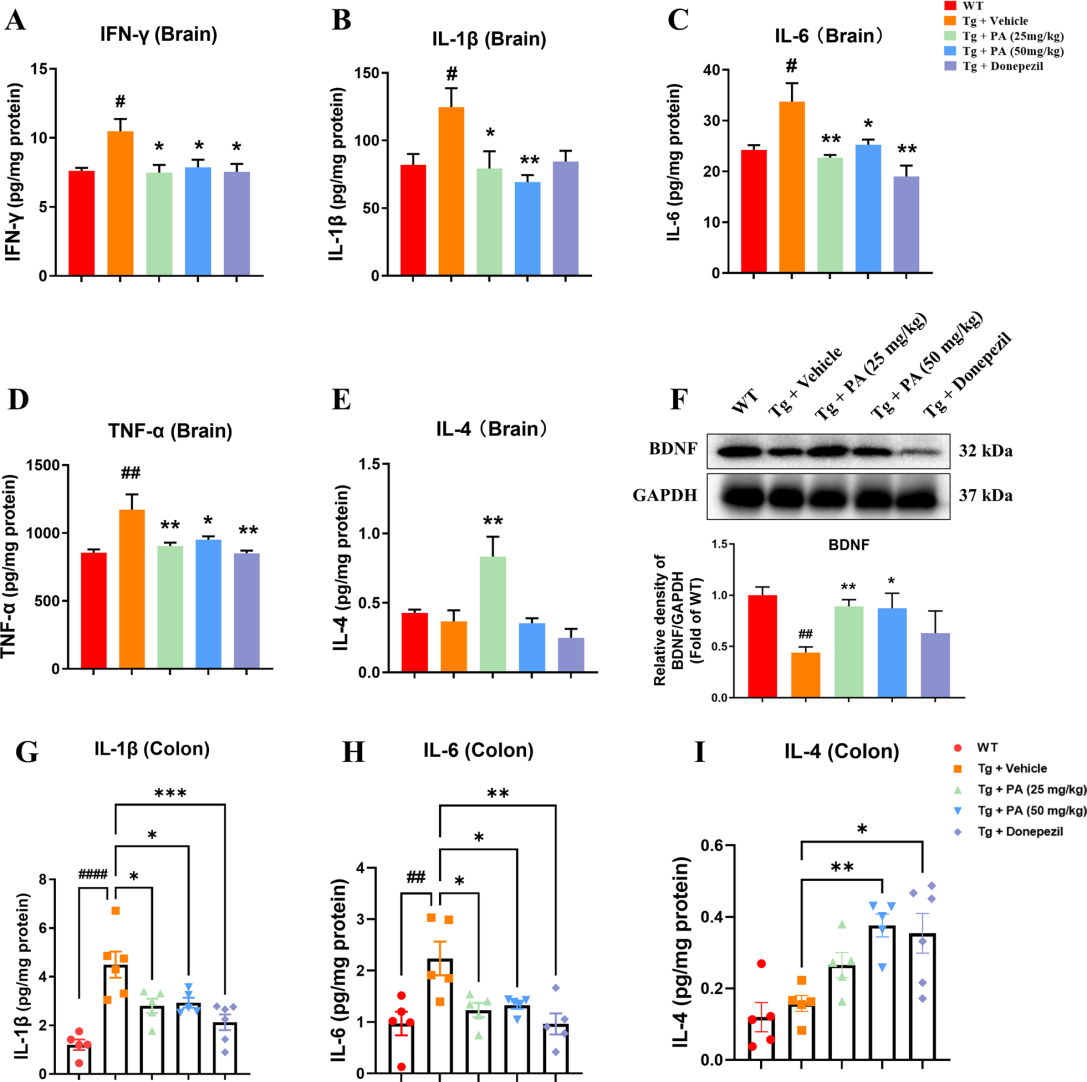
Effects of PA on pro-inflammatory cytokines, anti-inflammatory cytokines and BDNF in TgCRND8 mice. **A**–**E** Pro-inflammatory and anti-inflammatory cytokines in the brain. **A** IFN-γ; **B** IL-1β; **C** IL-6; **D** TNF-α; **E** IL-4; **F** the protein expression of BDNF; **G**–**I** pro-inflammatory and anti-inflammatory cytokines in the colon. **G** IL-1β; **H** IL-6; **I** IL-4. Data were expressed as the mean ± SEM (*n* = 5–6). ^##^*p* < 0.01 and ^####^*p* < 0.0001 compared with the WT group; *p < 0.05, **p < 0.01 and ***p < 0.001 compared with the Tg + vehicle group

As shown in Fig. 3A–D, the Tg + vehicle group had significantly higher levels of pro-inflammatory cytokines, including IFN-γ (*p* < 0.05), IL-1β (*p* < 0.05), IL-6 (*p* < 0.05) and TNF-α (*p* < 0.01), than the WT group. The administration of PA at the doses of 25 mg/kg and 50 mg/kg significantly decreased the elevated levels of IFN-γ (*p* < 0.05 for both), IL-1β (*p* < 0.05 and *p* < 0.01, respectively), IL-6 (*p* < 0.01 and *p* < 0.05, respectively) and TNF-α (*p* < 0.01 and *p* < 0.05, respectively) in the brain of TgCRND8 mice. On the other hand, donepezil treatment markedly reduced the levels of IFN-γ (*p* < 0.05), IL-6 (*p* < 0.01) and TNF-α (*p* < 0.01), when compared with the Tg control group. Interestingly, no significant difference was found in the level of anti-inflammatory cytokine IL-4 between the WT group and the Tg + vehicle group. However, the lower dose of PA (25 mg/kg) could significantly increase the IL-4 level (*p* < 0.01), when compared with the Tg control group (Fig. 3E).

In addition to its neuroprotective effects, BDNF was found to participate in anti-inflammatory processes and rescue hippocampal apoptosis [46]. Thus, the anti-inflammatory effects of PA were further determined by analyzing the protein expression of BDNF. As shown in Fig. 3F, there was a significant reduction in the protein expression of BDNF in the Tg + vehicle group (*p* < 0.01), when compared with WT group, and the decreased BDNF expression was effectively elevated upon PA (25 mg/kg and 50 mg/kg) treatment (*p* < 0.01 and *p* < 0.05, respectively), when compared with the Tg control group.

### Effects of PA on pro-inflammatory and anti-inflammatory cytokines in the colon tissues of TgCRND8 mice

The effects of PA on pro-inflammatory and anti-inflammatory cytokines in the colon tissues of TgCRND8 mice were generally congruent with that seen in the brain tissues (Fig. 3A–E). As shown in Fig. 3G–H, the Tg + vehicle group had significantly higher levels of IL-1β (*p* < 0.0001) and IL-6 (*p* < 0.01) than the WT group. The administration of PA at a dose of 25 mg/kg and 50 mg/kg efficiently reduced the elevated levels of IL-1β (*p* < 0.05 for both) and IL-6 (*p* < 0.05 for both) in the colon tissues of TgCRND8 mice, as compared with the Tg + vehicle group. Donepezil treatment also markedly decreased the levels of IL-6 (*p* < 0.001) and TNF-α (*p* < 0.01), when compared with the Tg control group. No significant difference was found in the level of the anti-inflammatory cytokine IL-4 between the WT group and the Tg + vehicle group. However, the high dose of PA (50 mg/kg) and donepezil (5 mg/kg) significantly increased the IL-4 level (*p* < 0.01 and* p* < 0.05, respectively), as compared with the Tg + vehicle group (Fig. 3I).

### Effects of PA on gut dysbiosis in TgCRND8 mice

#### Effects of PA on α-diversity and principal coordinate plot (PCoA) analysis in TgCRND8 mice

**Fig. 4:**
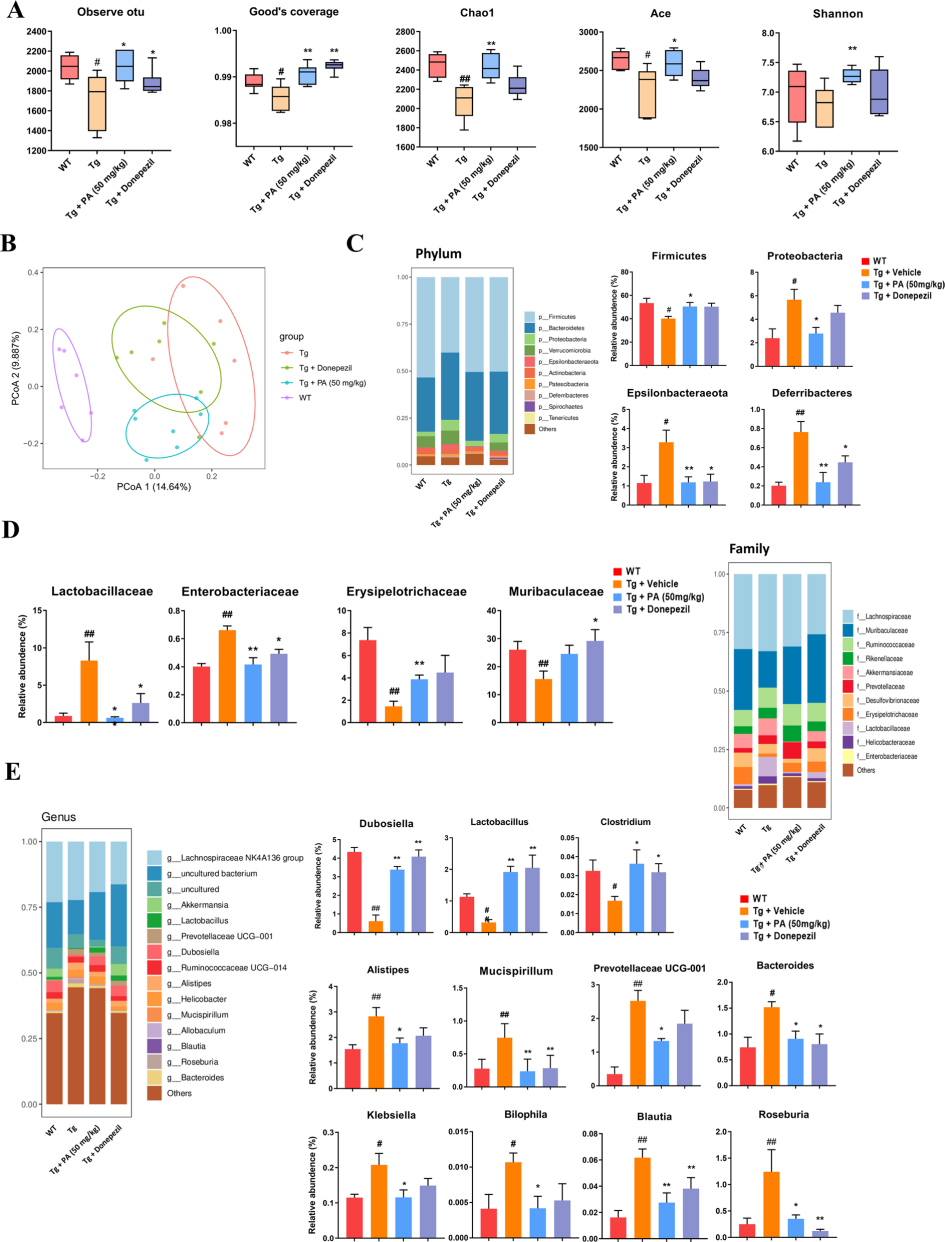
Effects of PA on gut dysbiosis in TgCRND8 mice. **A** Effects of PA on α-diversity in TgCRND8 mice. Observed OUT represents the actual observed number of operational taxonomic units (OTUs). Good's coverage reflects the depth of sequencing. The closer the Good's coverage is to 1, the deeper the sequencing has covered all species in the sample. Chao1 and ACE estimate microbiota’s total richness, that is, the number of species observed in each sample. Shannon index estimates both the richness and evenness of the microbiota community and provides information about microbial diversity indexes; **B** PCoA of microbial community structure; **C** gut microbiota composition at the phylum level; **D** gut microbiota composition at the family level; **E** gut microbiota composition at the genus level. Data were expressed as the mean ± SEM (*n* = 6). ^#^*p* < 0.05 and ^##^*p* < 0.01 compared with the WT group; *p < 0.05 and **p < 0.01 compared with the Tg + vehicle group

As shown in Fig. 4A, α-diversity analysis results displayed that TgCRND8 mice had significantly lower sequencing depth index and bacterial richness than WT mice, as demonstrated by marked decreased levels of observed operational taxonomic unit (OTU) (*p* < 0.05), Good’s coverage (*p* < 0.05), Chao1 (*p* < 0.01) and abundance-based coverage estimator (ACE) (*p* < 0.05) in TgCRND8 mice. PA (50 mg/kg) treatment significantly increased OTU (*p* < 0.05) and Good’s coverage (*p* < 0.01) in TgCRND8 mice as compared with the Tg control group, leading to restoration of the sequencing depth index. PA (50 mg/kg) treatment also significantly up-regulated Chao1 (*p* < 0.01) and ACE (*p* < 0.05) in TgCRND8 mice as compared with the Tg control group, indicating that PA could improve the deficits of the bacterial richness. No difference in the Shannon index was found between the WT group and the Tg control group. PA (50 mg/kg) treatment effectively increased the Shannon index (*p* < 0.01) in TgCRND8 mice, when compared with the Tg control group. Additionally, to display the differences of OTU, the dynamic alteration of the microbial community composition was assessed from PCoA (weighted Unifrac). As shown in Fig. 4B, the PCoA results indicated that the gut microbiota communities in the WT group were clustered away from that of the Tg group. Gut microbiota communities of the PA-treated TgCRND8 mice clustered distinctly and differed from that of the TgCRND8 control mice, suggesting that the gut microbiota communities were altered and tended to recover to normal after PA treatment.

#### Effects of PA on gut microbiota composition at the phylum level

Then gut microbiota distribution at different levels in the fecal samples was identified. As shown in Fig. 4C, at the phylum level, the proportion of *Firmicutes* in TgCRND8 mice was decreased by 24.8% (*p* < 0.05), while the abundances of *Proteobacteria*, *Epsilonbacteraeota* and *Deferribacteres* in TgCRND8 mice were increased by 136.8% (*p* < 0.05), 185.2% (*p* < 0.05) and 276.6% (*p* < 0.01), respectively, as compared with the WT mice. In addition, the alterations of *Firmicutes*, *Proteobacteria*, *Epsilonbacteraeota* and *Deferribacteres* were effectively restored by PA treatment (*p* < 0.05, *p* < 0.05, *p* < 0.01 and *p* < 0.01, respectively), when compared with the Tg control group.

#### Effects of PA on gut microbiota composition at the family level

At the family level, when compared with the WT group, the relative abundances of *Lactobacillaceae* and *Enterobacteriaceae* were significantly increased by 8.45-fold and 64.6% (*p* < 0.01 for both), respectively, while the proportions of *Erysipelotrichaceae* and *Muribaculaceae* were significantly decreased by 80.3% and 40.3% (*p* < 0.01 for both), respectively, in TgCRND8 mice. The alterations of *Lactobacillaceae*, *Enterobacteriaceae* and *Erysipelotrichaceae* were effectively restored by PA treatment (*p* < 0.05, *p* < 0.01 and *p* < 0.01, respectively), when compared with the Tg control group (Fig. 4D).

#### Effects of PA on gut microbiota composition at the genus level

At the genus level, when compared with the WT group, the relative abundances of *Dubosiella*, *Lactobacillus* and *Clostridium* were significantly attenuated by 85.8%, 71.6% and 48.4%, respectively, while the proportions of *Alistipes, Mucispirillum*, *Prebotellaceae UCG-001*, *Bacteroides*, *Klebsiella*, *Bilophila*, *Blautia* and *Roseburia* were significantly increased by 80.2%, 168.2%, 624.3%, 104.8%, 80.4%, 158.6%, 279.7% and 394.8%, respectively, in TgCRND8 mice. On the other hand, the alterations of *Dubosiella*, *Lactobacillus*, *Clostridium, Alistipes, Mucispirillum*, *Prebotellaceae UCG-001*, *Bacteroides*, *Klebsiella*, *Bilophila*, *Blautia* and *Roseburia* were effectively restored by PA treatment, when compared with the Tg control group (Fig. 4E).

### Effects of PA on the protein expressions of C/EBPβ and AEP in the hippocampal and colon tissues of TgCRND8 mice

**Fig. 5:**
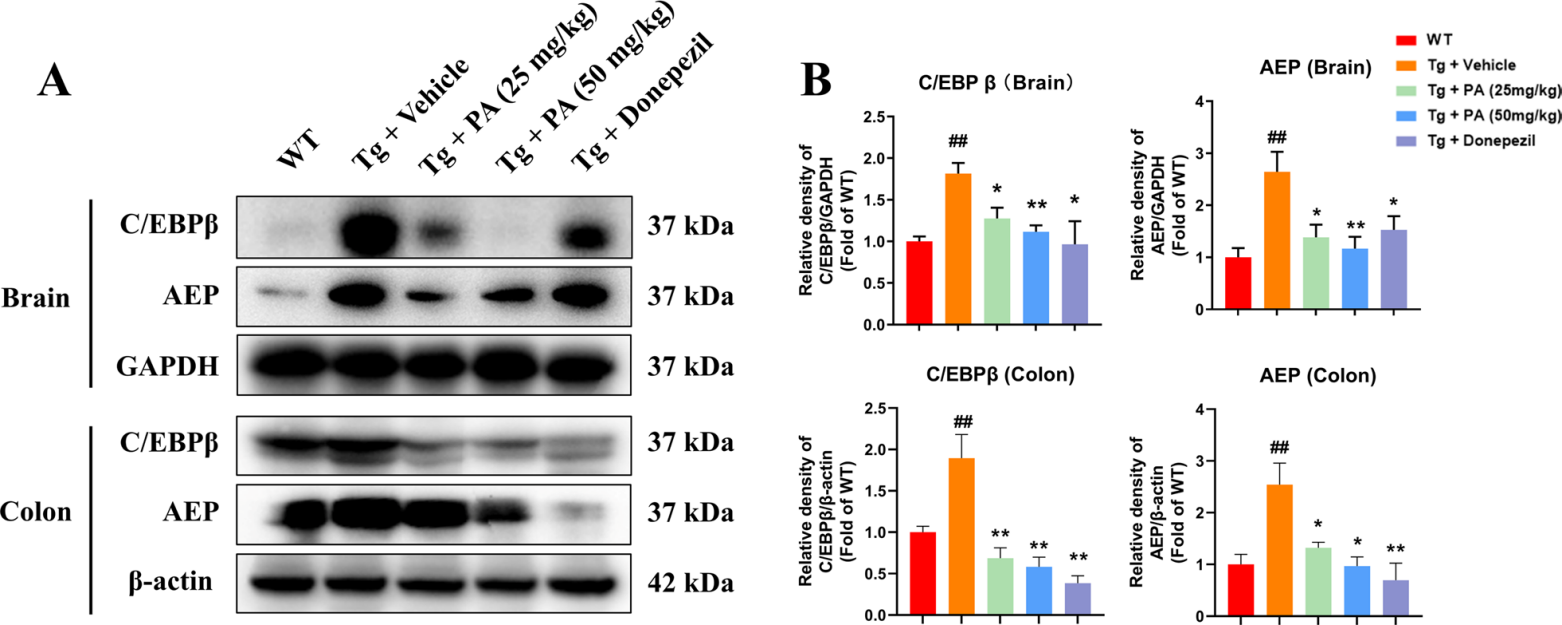
Effects of PA on the protein expressions of C/EBPβ and AEP in the hippocampus and colon of TgCRND8 mice. **A** Representative western blotting images; **B** quantitative analysis of the protein expressions of C/EBPβ and AEP. Data were expressed as the mean ± SEM (*n* = 3–4). ^##^*p* < 0.01 compared with the WT group; *p < 0.05 and **p < 0.01 compared with the Tg + vehicle group

The western blot results showed that the Tg + vehicle group had marked higher expressions of C/EBPβ (*p* < 0.01 for both hippocampal and colon tissues) and AEP (*p* < 0.01 for both hippocampal and colon tissues) than the WT group. Treatment with PA (25 mg/kg and 50 mg/kg) significantly down-regulated the protein expressions of C/EBPβ (*p* < 0.05 and *p* < 0.01, respectively) and AEP (*p* < 0.05 and *p* < 0.01, respectively) in the hippocampus of Tg mice, as compared with the Tg control group. Moreover, PA at the doses of 25 mg/kg and 50 mg/kg markedly decreased the protein expressions of C/EBPβ (*p* < 0.01 for both) and AEP (*p* < 0.05 for both) in the colon tissues of Tg mice. The administration of donepezil (5 mg/kg) also suppressed the levels of C/EBPβ (*p* < 0.05 for hippocampus and *p* < 0.01 for colon, respectively) and AEP (*p* < 0.05 for hippocampus and *p* < 0.01 for colon, respectively), when compared with the Tg control group (Fig. 5).

### Effects of PA on behavioral impairments, C/EBPβ/AEP signaling pathway and AD-like pathologies after the overexpression of C/EBPβ in the hippocampus of TgCRND8 mice

To further verify whether PA exerts anti-AD effects via modulating C/EBPβ/AEP signaling pathway in TgCRND8 mice, the C/EBPβ was overexpressed through AAV injection in the hippocampus of TgCRND8 mice. AAV, a member of the *Parvoviridae* family, is a class of icosahedral parvoviridae viruses with a diameter of about 20–26 nm and a genome of 4.7 kb linear single strand DNA. AAV has been widely used as one of the most promising gene transfer vectors for gene research and gene therapy due to its high safety, low immunogenicity, wide range of host cells (capable of infecting both dividing and non-dividing cells) and long-term gene expression in vivo [47–50]. The recombinant AAV used in this study is a gene vector modified from the non-pathogenic wild-type AAV.

#### Effects of PA on locomotor activity and the anxiety-related behavior after the overexpression of C/EBPβ in the hippocampus of TgCRND8 mice

**Fig. 6:**
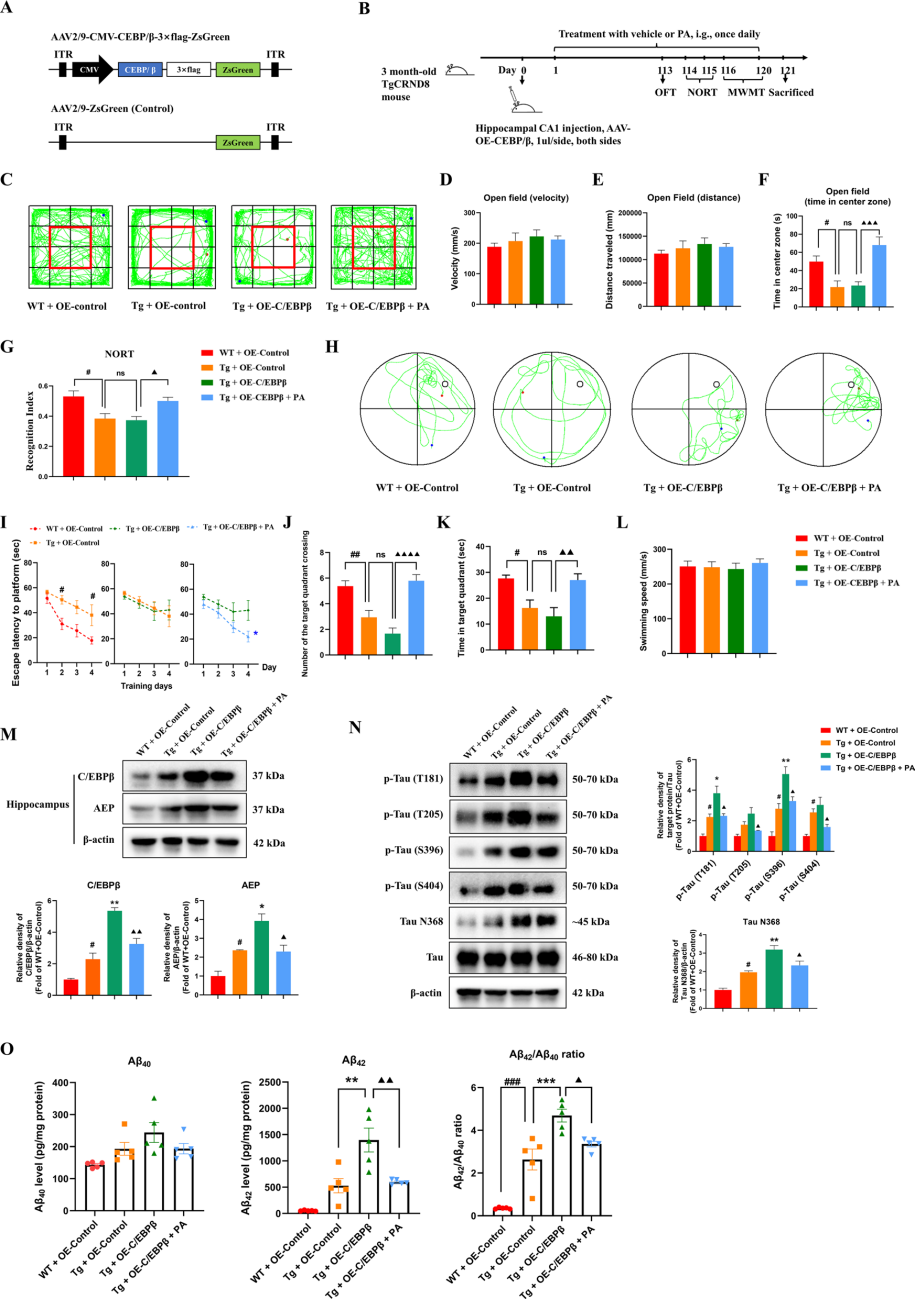
Effects of PA on behavioral impairments, C/EBPβ/AEP signaling pathway and AD-like pathologies after the overexpression of C/EBPβ in the hippocampus of TgCRND8 mice. **A** Schematic representation of the AAV genome encoding the CEBP/β sequence under the control of the CMV promoter; **B** experimental design and treatment schedule for identifying the potential mechanisms underlying the effects of PA on the TgCRND8 mice; **C**–**L** effects of PA (50 mg/kg) on behavioral impairments and cognitive deficits after the overexpression of C/EBPβ in the hippocampus of TgCRND8 mice (*n* = 8). **C** Representative images of the movement paths in OFT; **D** movement velocity in OFT; **E** distance traveled in OFT; **F** time spent in the center zone in OFT; **G** Recognition Index in NORT; **H** representative images of the swimming paths of mice in the MWMT probe test; **I** escape latency to platform during training days in MWMT; **J** numbers of the target crossing in the MWMT probe test; **K** the time spent in the target quadrant in the MWMT probe test; **L** swimming speed in the target quadrant in the MWMT probe test; **M** representative western blotting images and quantitative analysis of the protein expressions of C/EBPβ and AEP in the hippocampus (*n* = 3); **N** representative western blotting images and quantitative analysis of the protein expressions of hyperphosphorylated tau and Tau N368 in the hippocampus (*n* = 3); **O** Aβ_40_ and Aβ_42_ levels and Aβ_42_/Aβ_40_ ratio (*n* = 5). Data were expressed as the mean ± SEM (*n* = 3–8). ^#^*p* < 0.05, ^##^*p* < 0.01 and ^###^*p* < 0.001 compared with the WT + OE-Control group; *p < 0.05, **p < 0.01 and ***p < 0.001 compared with the Tg + OE-Control group; ^▲^*p* < 0.05, ^▲▲^*p* < 0.01 and ^▲▲▲▲^*p* < 0.0001 compared with the Tg + OE-C/EBPβ group

The behavioral tests were first conducted to determine the effect of PA on the neurological functions in TgCRND8 mice after AAV injection. As shown in Fig. 6D, E, there were no significant differences in distance traveled and movement velocity among all groups. Consistent with previous findings (Fig. 1H), there was a transgene-dependent reduction in the time exploring the center zone of the chamber; that is, mice in the Tg + OE-control group spent markedly less time exploring the center zone of the open field (*p* < 0.05) than that of the WT + OE-control group (Fig. 6F). However, no significant difference in the time exploring the center zone of the open field was found between the Tg + OE-control group and the Tg + OE-C/EBPβ group. The mice in the Tg + OE-C/EBPβ + PA group spent significantly more time exploring the center zone of the open field than that in the Tg + OE-C/EBPβ group (*p* < 0.001) (Fig. 6F). These findings suggested that AAV injection did not cause motor coordination deficits and the anxiety-related behavioral deficits in TgCRND8 mice. Moreover, PA could alleviate the anxiety-related behavioral deficits after AAV injection in the hippocampus of TgCRND8 mice.

#### Effects of PA on recognition memory after the overexpression of C/EBPβ in the hippocampus of TgCRND8 mice

As shown in Fig. 6G, the Tg + OE-control group had a markedly lower recognition index than the WT + OE-control group (*p* < 0.05), which is generally congruent with the previous experiment (Fig. 1J). No statistically significant difference was observed in the recognition index between the Tg + OE-control group and the Tg + OE-C/EBPβ group. Treatment with PA significantly up-regulated the recognition index (*p* < 0.05) after the overexpression of C/EBPβ in the hippocampus of TgCRND8 mice, when compared with the Tg + OE-C/EBPβ group.

#### Effects of PA on cognitive and learning functions after the overexpression of C/EBPβ in the hippocampus of TgCRND8 mice

The MWMT results showed that during the training days, the mice in the Tg + OE-control group spent significantly longer time finding the hidden platform than the WT + OE-control group (day 2, *p* < 0.05; day 4, *p* < 0.05). No significant difference was observed in the escape latency to the hidden platform between the Tg + OE-control group and the Tg + OE-C/EBPβ group. As compared with the Tg + OE-C/EBPβ group, the mice in the Tg + OE-C/EBPβ + PA group had a marked shorter escape latency to find the hidden platform during the training days (day 4, *p* < 0.05) (Fig. 6I).

In the probe trial, when compared with the WT + OE-control group, there were conspicuous reductions in the number of target quadrant crossing (*p* < 0.01) and the time spent in the target quadrant (*p* < 0.05) in the Tg + OE-control group (Fig. 6J, K), which was consistent with previous experimental results (Fig. 1K–O), indicating that Tg mice exhibit the cognitive and memory deficits. The mice in the Tg + OE-C/EBPβ group crossed the target quadrant less often and spent less time in the target quadrant than those in the Tg + OE-control group, though the differences were not statistically significant (Fig. 6J, K). PA treatment markedly increased the number of target quadrant crossing (*p* < 0.0001) and the time spent in the target quadrant (*p* < 0.01) after the overexpression of C/EBPβ in the hippocampus of TgCRND8 mice, as compared with the Tg + OE-C/EBPβ group (Fig. 6J, K). There were no statistical differences in the swimming speed among all groups (Fig. 6L).

#### Effects of PA on the protein expressions of C/EBPβ and AEP after the overexpression of C/EBPβ in the hippocampus of TgCRND8 mice

The protein expressions of C/EBPβ and AEP in the hippocampus were then determined. As shown in Fig. 6M, the Tg + OE-control group had significantly higher protein expressions of C/EBPβ (*p* < 0.05) and AEP (*p* < 0.05) than the WT + OE-control group, which were generally consistent with the previous finding (Fig. 5). AAV-C/EBPβ injection further up-regulated the protein levels of C/EBPβ (*p* < 0.01) and its downstream target AEP (*p* < 0.05) in the hippocampus of TgCRND8 mice, as compared with the Tg + OE-control group, indicating that AAV-C/EBPβ injection into the hippocampal CA1 region successfully induced the overexpression of C/EBPβ as expected. PA treatment markedly suppressed the elevated levels of C/EBPβ (*p* < 0.01) and AEP (*p* < 0.05) after the overexpression of C/EBPβ in the hippocampus of TgCRND8 mice, as compared with the Tg + OE-C/EBPβ group.

#### Effects of PA on the tau hyperphosphorylation and the protein expression of Tau N368 after the overexpression of C/EBPβ in the hippocampus of TgCRND8 mice

As shown in Fig. 6N, when compared with the WT + OE-control group, the protein levels of p-Tau (T181)/Tau, p-Tau (S396)/Tau and p-Tau (S404)/Tau were significantly increased (*p* < 0.05 for all) in the Tg + OE-control group. The Tg + OE-control group also had higher protein expression of p-Tau (T205)/Tau than the WT + OE-control group, but the difference was not statistically significant. AAV-C/EBPβ injection markedly elevated the relative ratios of p-Tau (T181)/Tau and p-Tau (S396)/Tau (*p* < 0.05 and *p* < 0.01, respectively) after the overexpression of C/EBPβ in the hippocampus of TgCRND8 mice, as compared with the Tg + OE-C/EBPβ group. No significant differences were found in the protein expressions of p-Tau (T205)/Tau and p-Tau (S404)/Tau between the Tg + OE-control group and the Tg + OE-C/EBPβ group, although the Tg + OE-C/EBPβ group did show a trend of up-regulation in the protein levels of p-Tau (T205)/Tau and p-Tau (S404)/Tau. PA treatment significantly reduced the protein levels of p-Tau (T181)/Tau, p-Tau (T205)/Tau, p-Tau (S396)/Tau and p-Tau (S404)/Tau (*p* < 0.05 for all) after the overexpression of C/EBPβ in the hippocampus of TgCRND8 mice, as compared with the Tg + OE-C/EBPβ group.

As shown in Fig. 6N, the Tg + OE-control group had significantly higher protein expression of Tau N368 (*p* < 0.05) than the WT + OE-control group, and this elevated protein level was further markedly up-regulated by AAV-C/EBPβ injection (*p* < 0.01). PA treatment significantly reduced the increased protein expression of Tau N368 (*p* < 0.05) after the overexpression of C/EBPβ in the hippocampus of TgCRND8 mice, as compared with the Tg + OE-C/EBPβ group.

#### Effects of PA on the levels of Aβ_40_ and Aβ_42_ after the overexpression of C/EBPβ in the hippocampus of TgCRND8 mice

As shown in Fig. 6O, when compared with the Tg + OE-control group, AAV-C/EBPβ injection further accentuated the elevated levels of Aβ_40_ (*p* > 0.05) and Aβ_42_ (*p* < 0.01) in TgCRND8 mice. PA treatment inhibited the increased levels of Aβ_40_ (*p* > 0.05) and Aβ_42_ (*p* < 0.01) after the overexpression of C/EBPβ in the hippocampus of TgCRND8 mice, when compared with the Tg + OE-C/EBPβ group.

Similarly, the Tg + OE-control group had a significant higher Aβ_42_/Aβ_40_ ratio than the WT + OE-control group (*p* < 0.001), and this elevated ratio was further up-regulated by AAV-C/EBPβ injection (*p* < 0.001). PA effectively suppressed the ratio of Aβ_42_/Aβ_40_ (*p* < 0.05) after the overexpression of C/EBPβ in the hippocampus of TgCRND8 mice, as compared with the Tg + OE-C/EBPβ group.

### Effects of PA-FMT on behavioral impairments, AD-like pathologies and C/EBPβ/AEP signaling pathway in TgCRND8 mice

PA was previously demonstrated to restore gut dysbiosis, alleviate intestinal inflammation and inhibit the activation of C/EBPβ/AEP signaling pathway in the colon (Figs. 3G–I, 4 and 5). To further identify the potential role of gut microbiota on the anti-AD effects of PA, FMT treatment was conducted.

#### Effects of PA-FMT treatment on the locomotor activity and the anxiety-related behavior in TgCRND8 mice

**Fig. 7:**
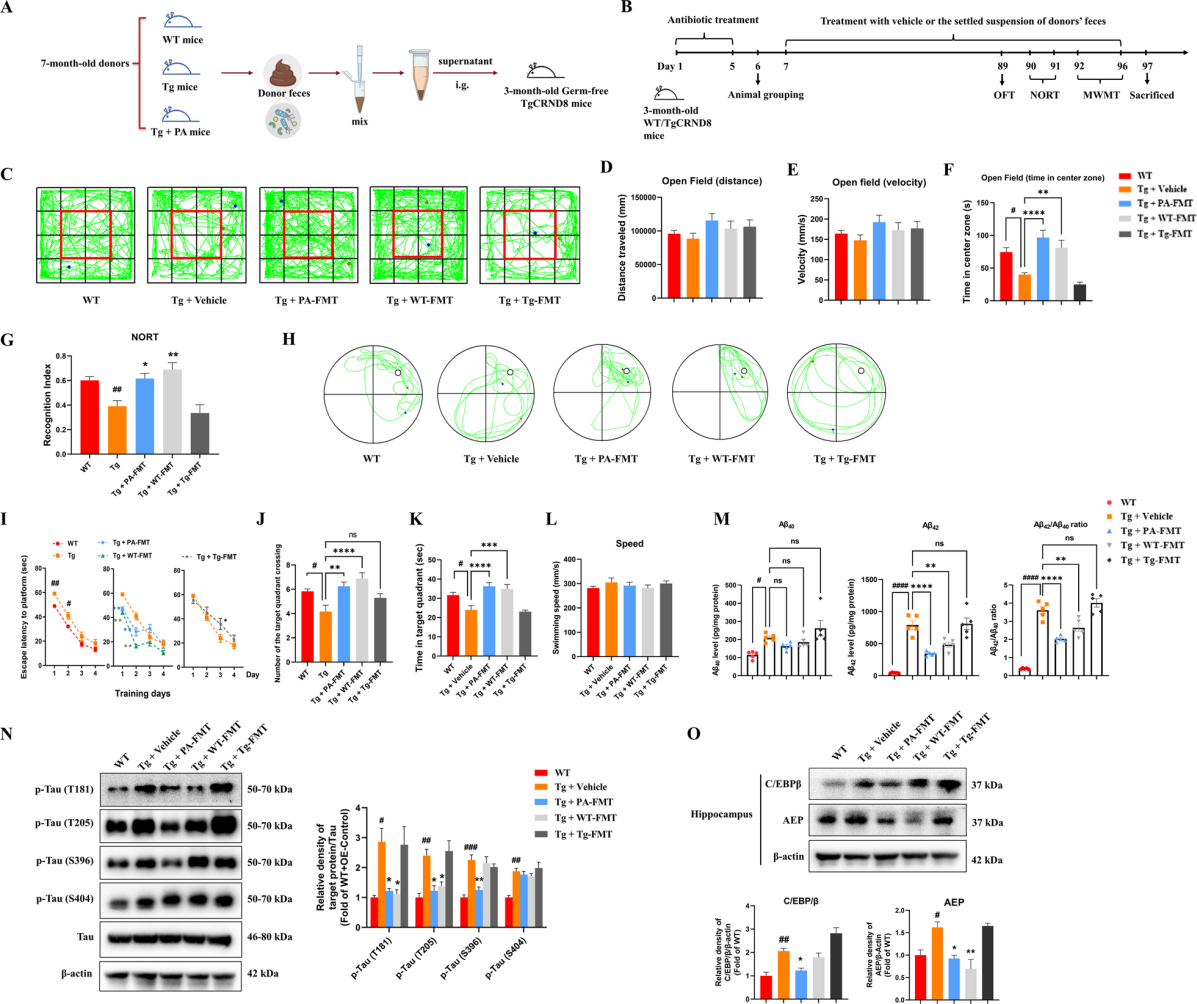
Effects of PA-FMT on behavioral impairments, AD-like pathologies and C/EBPβ/AEP signaling pathway in the germ-free TgCRND8 mice. **A**, **B** Experimental design and treatment schedule for identifying the involvement of gut microbiota on the anti-AD effects of PA; **C**–**K** effects of PA-FMT on behavioral impairments and cognitive deficits in the germ-free TgCRND8 mice (*n* = 7–8). **C** Representative images of the movement paths in OFT; **D** distance traveled in OFT; **E** movement velocity in OFT; **F** time spent in the center zone in OFT; **G** Recognition Index in NORT; **H** representative images of the swimming paths of mice in the MWMT probe test; **I** escape latency to platform during training days in MWMT; **J** number of the target quadrant crossing in the target quadrant in the MWMT probe test; **K** the time spent in the target quadrant in the MWMT probe test; **L** swimming speed in the MWMT probe test; **M** Aβ_40_ and Aβ_42_ levels and Aβ_42_/Aβ_40_ ratio (*n* = 5); **N** representative western blotting images and quantitative analysis of the protein expressions of hyperphosphorylated tau in the hippocampus (*n* = 3); **O** representative western blotting images and quantitative analysis of the protein expressions of C/EBPβ and AEP in the hippocampus (*n* = 3). Data were expressed as the mean ± SEM (*n* = 3–8). ^#^*p* < 0.05, ^##^*p* < 0.01 and ^####^*p* < 0.0001 compared with the WT group; *p < 0.05, **p < 0.01, ***p < 0.001 and ****p < 0.0001 compared with the Tg + vehicle group

The behavior tests were first conducted to determine the effect of FMT treatment on the neurological functions in TgCRND8 mice. As shown in Fig. 7D, E, OPT showed no significant differences in distance traveled and movement velocity among all groups, indicating that FMT treatment did not cause a motor coordination deficit. When compared with the WT mice, mice in the Tg + vehicle group spent markedly less time exploring the center zone of the open field (*p* < 0.05). The Tg mice with PA-FMT treatment and WT-FMT treatment spent significantly more time exploring the center zone than mice in the Tg + vehicle group (*p* < 0.0001 and *p* < 0.01, respectively) (Fig. 7F), indicating that PA-FMT treatment and WT-FMT treatment could alleviate the anxiety-related behavioral deficits. In addition, no significant difference in the time exploring the center zone of the open field was found between the Tg + vehicle group and the Tg + Tg-FMT group, although the Tg + Tg-FMT group did show a trend of reduction in the time exploring the center zone.

#### Effects of PA-FMT treatment on the recognition memory in TgCRND8 mice

The NORT was conducted to assess recognition memory in mice. As shown in Fig. 7G, when compared with the WT mice, mice in the Tg control group spent significantly less time exploring a novel object than the familiar one and had a much lower recognition index (*p* < 0.01). PA-FMT and WT-FMT markedly improved the recognition index (*p* < 0.05 and* p* < 0.01, respectively), as compared with the Tg + vehicle group, indicating that PA-FMT and WT-FMT could improve the recognition memory deficits in the germ-free TgCRND8 mice. No statistically significant difference was observed in the recognition index between the Tg control group and the Tg + Tg-FMT group, but the recognition index in the Tg + Tg-FMT group showed a slight downward trend, when compared the Tg + vehicle group.

#### Effects of PA-FMT treatment on cognitive and learning functions in TgCRND8 mice

The MWMT was then applied to assess the cognitive and learning functions in mice. As shown in Fig. 7I, during the training days, the mice in the Tg control group spent more time to find the hidden platform than those in the WT group (day 1, *p* < 0.01; day 2, *p* < 0.05; day 3 and day 4, *p* > 0.05 for both). As compared with the Tg control group, the mice in the Tg + PA-FMT group and the Tg + WT-FMT group had shorter escape latencies to find the hidden platform (day 1, *p* < 0.01 for both; day 2, *p* < 0.01 for both). However, the germ-free TgCRND8 mice with Tg-FMT treatment had markedly longer escape latencies to find the platform (*p* < 0.05 on day 3).

In the probe trial, when compared with the WT group, there were conspicuous reductions in the number of the target quadrant crossing and the time spent in the target quadrant in the Tg control group (*p* < 0.05 for both) (Fig. 7J, K). The germ-free TgCRND8 mice with PA-FMT treatment and WT-FMT treatment crossed the target quadrant more often (*p* < 0.01 and *p* < 0.0001, respectively) and spent significantly more time exploring in the target quadrant (*p* < 0.0001 and *p* < 0.001, respectively) than the mice in the Tg control group. In addition, the administration of Tg-FMT reduced the number of the target quadrant crossing and the time spent in the target quadrant in germ-free TgCRND8 mice, but the differences were not statistically significant (*p* > 0.05 for both), as compared with Tg control group. There were no statistical differences in the swimming speed among all groups (Fig. 7L). These experimental results indicated that PA-FMT could ameliorate the cognitive and learning impairments in the germ-free TgCRND8 mice.

#### Effects of PA-FMT treatment on the levels of Aβ_40_ and Aβ_42_ in the hippocampus of TgCRND8 mice

As shown in Fig. 7M, the level of Aβ_40_ in the Tg + vehicle group was significantly increased (*p* < 0.05), as compared with the WT group. When compared with the Tg + vehicle group, the administration of PA-FMT and WT-FMT decreased the Aβ_40_ level while Tg-FMT increased the Aβ_40_ level in the hippocampus of TgCRND8 mice, but the differences were not statistically significant (*p* > 0.05 for all).

Moreover, when compared with the WT group, the Aβ_42_ level and the Aβ_42_/Aβ_40_ ratio in the Tg + vehicle group were significantly increased (*p* < 0.0001 for both). PA-FMT treatment and WT-FMT treatment markedly attenuated the level of Aβ_42_ (*p* < 0.0001 for both) and the ratio of Aβ_42_/Aβ_40_ (*p* < 0.01 for both), when compared with the Tg + vehicle group. No significant differences in the Aβ_42_ level and the Aβ_42_/Aβ_40_ ratio were found between the Tg + vehicle group and the Tg + Tg-FMT group, but the Tg + Tg-FMT group had a non-significant higher ratio of Aβ_42_/Aβ_40_ than the Tg + vehicle group.

#### Effects of PA-FMT treatment on the hyperphosphorylation of tau protein in the hippocampus of TgCRND8 mice

As shown in Fig. 7N, when compared with the WT group, the relative ratios of p-Tau (T181)/Tau (*p* < 0.05), p-Tau (T205)/Tau (*p* < 0.01), p-Tau (S396)/Tau (*p* < 0.001) and p-Tau (S404)/Tau (*p* < 0.01) were significantly increased in the Tg control group. PA-FMT treatment markedly inhibited the hyperphosphorylation of tau protein at the sites of T181 (*p* < 0.05), T205 (*p* < 0.01) and S396 (*p* < 0.01), as compared with the Tg control group. Additionally, WT-FMT treatment significantly reduced the protein levels of p-Tau (T181)/Tau and p-Tau (T205)/Tau (*p* < 0.05 for both), as compared with the Tg control group.

#### Effects of PA-FMT treatment on the protein expressions of C/EBPβ and AEP in the hippocampus of TgCRND8 mice

As shown in Fig. 7O, the Tg control group had significantly higher protein expressions of C/EBPβ (*p* < 0.01) and AEP (*p* < 0.05) than the WT group. After FMT treatment, the protein levels of C/EBPβ and AEP were significantly decreased (*p* < 0.05 for both) in the Tg + PA-FMT group, as compared with the Tg control group. The administration of WT-FMT markedly reduced the protein expression of AEP (*p* < 0.01) in the hippocampus of TgCRND8 mice, as compared with the Tg group. In addition, the Tg + WT-FMT group had a non-significant higher protein expression of C/EBPβ (*p* > 0.05) than the Tg group. No differences were observed in the protein levels of C/EBPβ and AEP between the Tg + Tg-FMT group and the Tg group.

## Discussion

AD patients often suffer from progressive memory loss and severe cognitive dysfunction, accompanied by neuropsychiatric symptoms and behavior changes, such as anxiety, agitation, depression, irritability, disinhibition, aberrant motor behavior and sleep disturbances [51–54]. In line with the clinical findings, cognitive declines, behavioral abnormalities and neuropsychiatric changes were also observed in the AD animal models [55, 56]. In the present study, several behavioral tests, including BT, OFT, NORT and MWMT, were applied to assess different aspects of the neurological functions of TgCRND8 mice. All mice were tested on the BT to measure the ADL. Burrowing is a species-specific and spontaneous behavior largely dependent on the integrity of the hippocampus [43, 57], and has been found to be equivalent to ADL skills in humans [58]. The impairment of ADL skills is one of the pathological changes in AD patients [59–61], and has also been found in the AD preclinical models [62, 63]. Consistent with these findings, the present study showed that the TgCRND8 mice burrowed markedly less weight of food pellets than the WT mice, indicating that TgCRND8 mice have a significant deficit in spontaneous burrowing behavior. Besides, OFT was conducted in the present study to determine the locomotor activity and the anxiety level of the mice. Hyperactivity indicates agitation and restlessness in mice, but evidence regarding the activity levels in AD transgenic models is inconsistent, with the results suggesting either hyperactivity [64, 65], hypoactivity [66, 67], or no change [68, 69] in transgenic mice, as compared with their wild-type controls. The present study showed no significant differences in distance traveled and movement velocity among all groups in the OFT, indicating that TgCRND8 mice did not have motor coordination impairment at 7 months of age. In addition, OFT results also revealed that TgCRND8 mice spent less time exploring the center of the open field than the WT mice, suggesting that TgCRND8 mice have increased anxiety levels. Moreover, NORT was performed in this study to assess the recognition memory in mice, while MWMT was used to examine hippocampal-dependent learning and spatial memory. We found that the TgCRND8 mice had markedly lower recognition index than the WT mice in the NORT, while crossing the target quadrant less often and spending significantly less time in the target quadrant than the WT mice in the MWMT, indicating that TgCRND8 mice exhibited recognition memory impairments as well as learning and spatial memory deficits. PA treatment could significantly improve ADL, alleviate anxiety-related behavior deficits, and attenuate recognition memory and cognitive impairments in TgCRND8 mice. All these findings amply highlight the potential of PA as a therapeutic agent for AD.

The formation of amyloid plaques, mainly consisting of Aβ peptide, is one of the pathological hallmarks in the AD brain. The amyloid cascade hypothesis indicates that Aβ accumulates at the initial stage of AD, and subsequently aggregates in the brain, eventually leading to neurodegeneration [22, 70, 71]. It has been demonstrated that not only soluble oligomeric Aβ, but also the deposited Aβ in amyloid plaques can interact with microglia, astroglia, neurons and blood vessels, resulting in a series of detrimental cellular responses, ultimately causing neuronal death [72]. As an APP transgenic mouse model, TgCRND8 mice have an early onset of extracellular thioflavin S-positive Aβ deposits at 3 months of age, with dense-core Aβ plaques appearing from 5 months old [34]. The present study showed that PA could regulate the APP processing by reducing the protein expressions of β-secretase enzyme BACE-1 and γ-secretase enzymes such as APH-1 and p-APP (Thr688). PA could also increase the expression of the major Aβ-degrading enzyme IDE to regulate APP elimination. Moreover, PA significantly decreased both Aβ_40_ and Aβ_42_ levels, the two major Aβ peptides preceding Aβ plaque deposition, in the hippocampus of TgCRND8 mice. The immunofluorescence staining results showed that TgCRND8 mice had a larger number of and bigger Aβ plaques in the hippocampus and cortex than the WT controls, and this transgene-dependent increase in Aβ plaque burdens was effectively suppressed by PA treatment. All these experimental findings demonstrated that the neuroprotective properties of PA in AD are partly attributable to its suppressive effects on Aβ production and accumulation.

NFT, principally composed of hyperphosphorylated tau protein, is another pathological hallmark of AD, making a significant contribution to AD pathology [4]. The progression of AD is closely associated with abnormal hyperphosphorylation and aggregation of tau protein, while tau hyperphosphorylation has been observed in AD patients and is highly correlated with symptom severity [4, 73]. Phosphorylated tau at the site of Thr181 is recently recognized as a novel biomarker for AD diagnosis [74], while the hyperphosphorylation of tau protein at other sites, such as Thr205, Ser396 and Ser404, is also closely correlated with AD pathology [75]. The abnormal processing of tau pathology, especially the hyperphosphorylation and aggregation of tau protein, often appears from 7 months of age in the TgCRND8 mice [35]. The present study showed that PA treatment could markedly suppress the hyperphosphorylation of tau protein at the sites of Thr181, Thr205, Ser396 and Ser404 in TgCRND8 mice, indicating that the effect of PA on hyperphosphorylation of tau protein is one of the mechanisms underlying its cognitive improving properties.

Apart from amyloid plaques and NFTs, neuroinflammation also contributes to AD pathology, and participates in not only the onset but also the progression of AD [76–78]. Neuroinflammation is mainly mediated by central glial cells, such as astrocytes and microglia [79, 80]. In AD, microglia and astrocytes are usually activated by the Aβ accumulation, subsequently releasing pro-inflammatory cytokines, which further exacerbates tau hyperphosphorylation and aggravates Aβ deposition [81, 82]. Mounting evidence has indicated that pro-inflammatory cytokines are elevated in the brains of both AD patients and preclinical AD models, together with the activated microglia and astrocytes [23–25, 83]. Our present study showed significantly increased densities of Iba-1-positive microglia and GFAP-positive astrocytes in both the hippocampus and the cortex in the TgCRND8 mice, as compared with the WT mice. PA treatment effectively diminished the hyperactivation of microglia and astrocytes in the brains of TgCRND8 mice. The administration of PA also decreased the elevated levels of pro-inflammatory cytokines (IL-1β, IL-6, TNF-α and IFN-γ) and increased the anti-inflammatory cytokine IL-4. In addition, PA was found to significantly up-regulated the protein level of BDNF in TgCRND8 mice, as BDNF is known to participate in anti-inflammatory processes and rescue hippocampal apoptosis [46]. These results strongly suggest that the anti-AD effect of PA is, at least partially, associated with its anti-inflammatory property.

Emerging evidence has implied that abnormal gut microbiota is closely associated with AD progression [84, 85]. In the present study, the α-diversity analysis showed a reduced microbial community diversity in TgCRND8 mice (Fig. 4A), as demonstrated by marked decreased bacterial richness and sequencing depth index in TgCRND8 mice. Moreover, the dynamic alteration in the gut microbiota composition was evident from the PCoA results (Fig. 4B), which demonstrated a significant difference in the taxonomic distribution of the microbial community between the TgCRND8 mice and the WT mice. These findings were generally congruent with the decline of gut microbiota diversity in AD patients and 5 × FAD mice [8, 10, 86]. The present study showed that PA treatment effectively restored the sequencing depth index, bacterial richness, and the altered taxonomic distribution of the microbial community in TgCRND8 mice.

Furthermore, we also found that the relative abundance of *Firmicutes, Lactobacillus* and *Clostridium* in TgCRND8 mice was decreased by 24.8%, 71.6% and 48.4%, respectively, while the proportions of *Proteobacteria*, *Deferribacteres, Lactobacillaceae, Enterobacteriaceae, Alistipes, Bacteroides, Bilophila* and *Blautia* were increased by 136.8%, 276.6%, 845%, 64.6%, 80.2%, 104.8%, 158.6% and 279.7%, respectively, in TgCRND8 mice. In addition, the alterations of *Firmicutes, Lactobacillus, Clostridium, Proteobacteria*, *Deferribacteres, Lactobacillaceae, Enterobacteriaceae, Alistipes, Bacteroides, Bilophila* and *Blautia* were effectively restored by PA treatment. The phylum abundance of *Firmicutes* was decreased in AD patients [8, 87], whereas *Proteobacteria* was more abundant in AD patients [87] and APP/PS1 mice at six months of age [88]. Moreover, a reduction in *Firmicutes* has also been found in patients with type 2 diabetes mellitus (T2DM) and individuals with obesity [89, 90]. Notably, T2DM, insulin resistance and high body weight markedly increase the risk of developing AD [91–93]. Nagpal et al*.* [94] found that *Proteobacteria* was positively associated with the ratio of Aβ_1-42_/Aβ_1-40_ in elderly people with mild cognitive impairment (MCI), as compared with the age-matched control participants. In line with our results, higher relative abundances of *Lactobacillaceae* [10] and *Enterobacteriaceae* [87] were previously reported in AD patients. At the genus level, a lower proportion of *Clostridium* and higher proportions of *Alistipes, Bacteroides, Bilophila* and *Blautia* were found in AD patients [8]. Interestingly, the alterations of these gut microbiota at the genus level were closely associated with some CSF biomarkers of AD pathology, such as Aβ_42_/Aβ_40_ and hyperphosphorylated tau protein [95]. All these findings suggested that the inhibitory effects of PA on gut dysbiosis may be one of the mechanisms underlying its cognitively improving property in TgCRND8 mice.

Abnormal gut microbiota can cause intestinal inflammation and intestinal epithelial barrier dysfunction, leading to an increase in intestinal permeability and an aberrant reciprocal microbiota–gut–brain axis [84, 85]. Thus, the effects of PA on pro-inflammatory and anti-inflammatory cytokines in the colon of TgCRND8 mice were determined in the present study. Our results demonstrated that PA treatment could effectively decrease the elevated levels of pro-inflammatory cytokines such as IL-1β and IL-6, and increase the anti-inflammatory cytokine IL-4 in the colon tissues of TgCRND8 mice. Conceivably, the suppressive effects of PA on intestinal inflammation result from the alleviation of gut dysbiosis after PA treatment, specifically, the inhibitory effects of PA on the pro-inflammatory microbiota, such as *Bacteroides*, *Klebsiella*, *Bilophila*, *Proteobacteria* and *Enterobacteriaceae*, and the enhanced property of PA on the anti-inflammatory microbiota, including *Firmicutes* and *Lactobacillus*.

To further identify the involvement of gut microbiota on the anti-AD effects of PA, FMT treatment was conducted in this study. FMT is now widely applied as a potential therapeutic for many diseases [96, 97], especially for recurrent *Clostridium difficile* infection (rCDI), with a clinical success rate of more than 90% [98, 99]. Gut microbiota composition following FMT is restored to a state similar to the healthy donor [100, 101]. Kim et al*.* [102] found that the transplantation of healthy microbiota would ameliorate amyloid and tau pathologies in a germ-free transgenic mouse model of AD. Similar findings were reported in another AD animal model. The transplantation of germ-free APP/PS1 transgenic mice with microbiota from WT mice ameliorated cerebral amyloid pathology, while the transplantation of microbiota from conventionally raised APP/PS1 mice accentuated cerebral Aβ burden [14]. Our present study demonstrated that the FMT of fecal microbiota from both PA-treated TgCRND8 mice and WT mice substantially alleviated the impairments of anxiety-related behavior, recognition memory, and learning and spatial memory in the germ-free TgCRND8 mice. In addition, the administrations of PA-FMT and WT-FMT markedly inhibited hyperphosphorylation of tau protein and decreased Aβ level in the brain tissues of TgCRND8 mice. However, the FMT of microbiota from 7-month-old TgCRND8 mice did not exacerbate the cognitive dysfunctions and AD-like pathogenesis as expected. There were generally no differences in the behavioral deficits and AD-like pathogenesis between the Tg-FMT-treated TgCRND8 mice and vehicle-treated TgCRND8 mice. The reason may lie in two aspects: firstly, the treatment period may not be long enough to cause significant differences; secondly, since gut dysbiosis worsens with age [12, 103], the donors of fecal microbiota maybe not be old enough to accentuate the AD-like pathogenesis in the germ-free TgCRND8 mice. All these findings implicate that the modulatory effects of PA on gut dysbiosis contribute to the anti-AD effects of PA.

C/EBPβ/AEP signaling pathway is closely associated with AD pathologies [26]. The present study showed that TgCRND8 mice had significantly higher expressions of C/EBPβ and AEP in the hippocampus than the WT mice, and these elevated protein levels were reversed upon PA treatment, indicating that the anti-AD effects of PA may be mediated via inhibiting the activation of C/EBPβ/AEP signaling pathway. To further confirm whether PA exerts neuroprotective effects via modulating C/EBPβ/AEP signaling pathway in TgCRND8 mice, the C/EBPβ was overexpressed mediated by AAV injection in the hippocampus of TgCRND8 mice. We found that the overexpression of C/EBPβ resulted in an increase of AEP expression, as compared with the control virus-treated TgCRND8 mice, while the control virus-treated TgCRND8 mice still expressed higher levels of AEP in the hippocampus than the control virus-treated WT mice. Strikingly, PA treatment suppressed this C/EBPβ-elicited increase in AEP expression. Furthermore, the overexpression of C/EBPβ enhanced the expression of AEP-cleaved tau N368 proteolytic fragments, tau hyperphosphorylation and Aβ level to a greater extent than that seen with control virus-treated TgCRND8 mice, and all these AD-like pathogenesis induced by C/EBPβ were substantially ameliorated upon PA treatment.

It has been recently revealed that gut dysbiosis is correlated with the activation of the C/EBPβ/AEP signaling pathway in the gut with age in 5 × FAD mice [86]. Our present study showed that PA treatment significantly down-regulated the elevated protein expressions of C/EBPβ and AEP in the colon of TgCRND8 mice, indicating that PA could suppress the activation of the C/EBPβ/AEP signaling pathway in the colon of TgCRND8 mice. Moreover, after FMT treatment, the protein levels of C/EBPβ and AEP were significantly decreased in the brains of the PA-FMT-treated TgCRND8 mice, as compared with the vehicle-treated TgCRND8 control mice, which further demonstrated that PA could exert anti-AD effects by modulating gut microbiota via inhibition of the activation of C/EBPβ/AEP signaling pathway in TgCRND8 mice. Altogether, the present study demonstrated that the anti-AD effects of PA were mediated via C/EBPβ/AEP signaling pathway in TgCRND8 mice.

## Conclusions

**Fig. 8:**
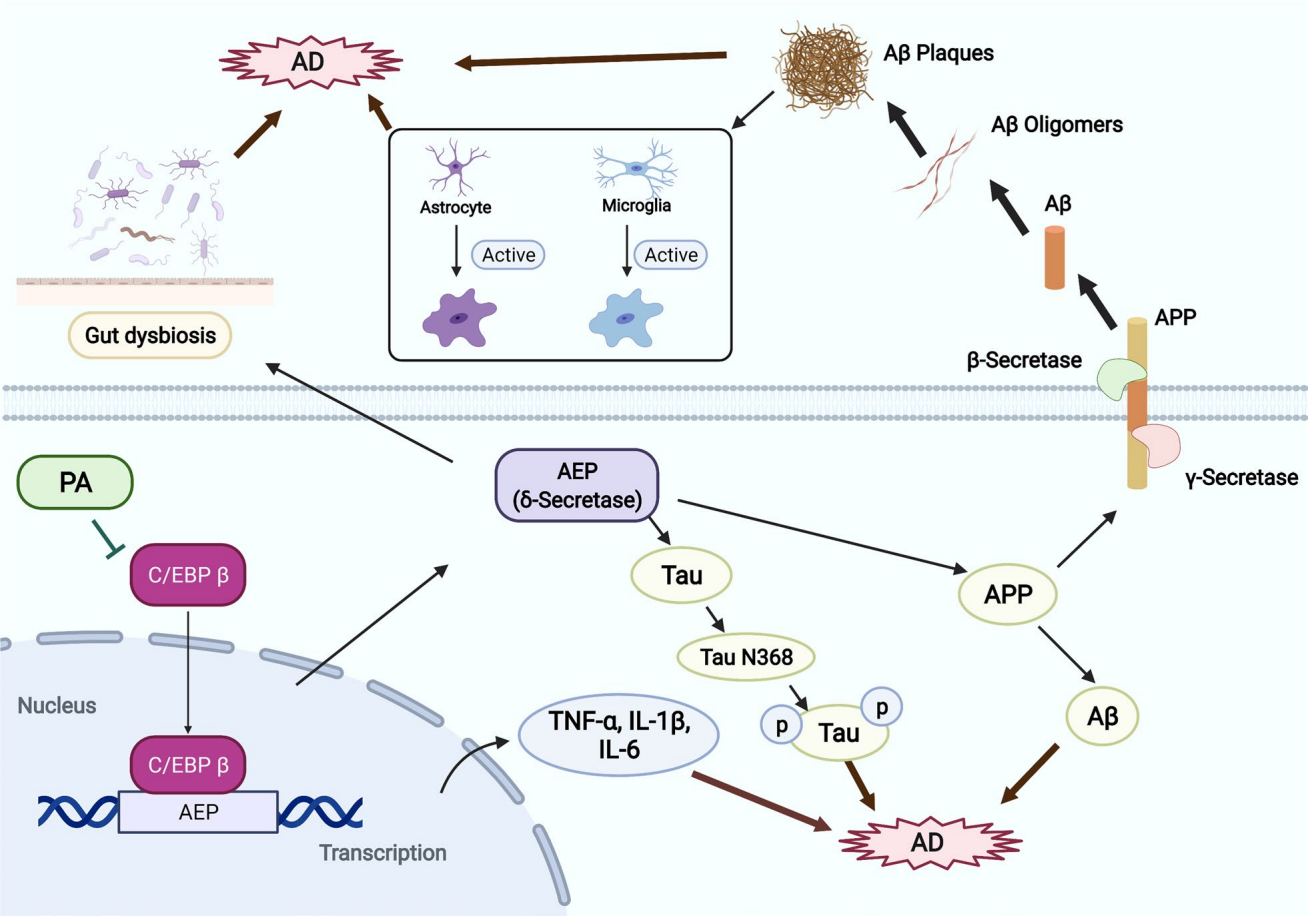
The schematic diagram summarizing the proposed mechanisms underlying the neuroprotective effects of PA in TgCRND8 mice

To conclude, the present study has amply demonstrated that PA could ameliorate the cognitive deficits in TgCRND8 mice via suppressing Aβ plaques deposition, the hyperphosphorylation of tau protein, neuroinflammation and gut dysbiosis through inhibiting the activation of C/EBPβ/AEP pathway (Fig. 8), suggesting that PA is a potential candidate worthy of further development into the pharmaceutical treatment of AD.

## Data Availability

All data supporting the conclusions of this article are included with this article.

## Abbreviations

AAV: Adeno-associated virus
Aβ: Beta-amyloid
ABX: Antibiotic cocktail
ACE: Abundance-based coverage estimator
AD: Alzheimer’s disease
ADL: Activities of daily living
AEP: Asparagine endopeptidase
APH-1: Anterior pharynx-defective-1
APP: Amyloid precursor protein
BT: Burrowing test
C/EBPβ: CCAAT/enhancer-binding protein beta
CNS: Central nervous system
DAPI: 4,6-Diamidino-2-phenylindole
ECL: Enhanced chemiluminescence
FMT: Fecal microbial transplantation
GI: Gastrointestinal
IBD: Inflammatory bowel disease
IBS: Irritable bowel syndrome
IDE: Insulin degrading enzyme
IL-1β: Interleukin-1β
MWMT: Morris water maze test
NFT: Neurofibrillary tangle
NORT: Novel object recognition test
OE: Overexpression
OFT: Open field test
PA: Patchouli alcohol
PBS: Phosphate-buffered saline
PBST: Phosphate-buffered saline with Tween^®^ 20
PCR: Polymerase chain reaction
PCoA: Principal coordinate analysis
PFA: Paraformaldehyde
PVDF: Polyvinyl difluoride
TNF-α: Tumor necrosis factor alpha
WT: Wild-type
